# Polychlorinated Diphenyl Ethers in the Environment: A Review and Future Perspectives

**DOI:** 10.3390/ijerph20053982

**Published:** 2023-02-23

**Authors:** Qiuxuan Wu, Qiong Wu, Xiaoxiang Wang, Xuesheng Zhang, Rui Zhang

**Affiliations:** 1 School of Water Conservancy and Environment, University of Jinan, Jinan 250022, China; 2 Institute for Carbon-Neutral Technology, Shenzhen Polytechnic, Shenzhen 518055, China; 3 School of Resources and Environmental Engineering, Anhui University, Hefei 230601, China; 4 Laboratory of Wetland Protection and Ecological Restoration, Anhui University, Hefei 230601, China

**Keywords:** PCDEs, persistent substances, bioaccumulation, biomagnification, environmental behavior, environmental fate

## Abstract

Polychlorinated diphenyl ethers (PCDEs) are a class of synthetic halogenated aromatic compounds, which have gradually attracted widespread attention due to potential environmental risks to humans and ecosystems. This paper presents a literature review of research on PCDEs using PubMed, Web of Science and Google Scholar as search engines/databases with no constraints on publishing year or number. A total of 98 publications on the sources, environmental levels, environmental behavior and fate, synthesis and analysis and toxicology of PCDEs were retrieved. Existing studies have shown that PCDEs widely exist in the environment with the ability of long-range transport, bioaccumulation and biomagnification, which are almost comparable to polychlorinated biphenyls. They can elicit adverse effects including hepatic oxidative stress, immunosuppression, endocrine disorders, growth retardation, malformations, reduced fertility and increased mortality in organisms, among which some seem to be related to the activation of the aryl hydrocarbon receptor. PCDEs can be metabolized into other organic pollutants, such as hydroxylated and methoxylated PCDEs and even polychlorinated dibenzo-*p*-dioxins and furans through biotransformation, photolysis and pyrolysis reactions in the environment. Compared with reviews on PCDEs published previously, some new information and findings are summarized in this review, such as new sources, current environmental exposure levels, main metabolism pathways in aquatic organisms, acute toxicity data for more species and relationships between structural parameters and toxicity and bioaccumulation potentials of PCDE congeners. Finally, current research deficiencies and future research perspectives are proposed to facilitate the assessment of health and ecological risks of PCDEs.

## 1. Introduction

**Figure 1:**
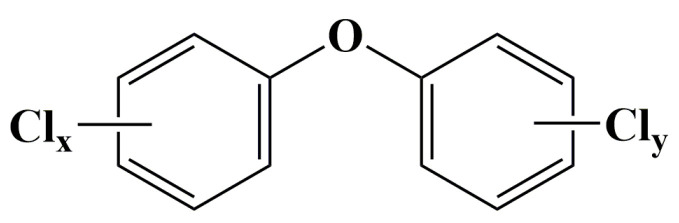
General chemical structure of polychlorinated diphenyl ethers (x + y ≤ 10).

Polychlorinated diphenyl ethers (PCDEs) are a class of synthetic halogenated aromatic compounds comprising 209 possible congeners, which are structurally similar to polychlorinated biphenyls (PCBs) and polychlorinated dibenzo-*p*-furans (PCDFs) [1]. However, PCDEs are typically more polar than PCBs due to the presence of the oxygen atom and the resultant asymmetry over the horizontal axis [2,3]. The theoretical 209 congeners can be divided into ten congener groups from mono- to deca-CDE and numbered (Table S1) according to the International Union of Pure and Applied Chemistry (IUPAC) system established for PCBs [3]. The structural formula of PCDEs is shown in Figure 1 and their molecular formula is C_12_H_10–n_Cl_n_O (n = 1–10).

PCDEs were widely used as flame retardants, hydraulic fluids, electric insulators, lubricants and plasticizers in the 20th century [4,5]. Currently, congener CDE 13 is used directly as the intermediate in the synthesis of the fungicide difenoconazole [6]. In addition, PCDEs are by-products produced during the synthesis of commercial chlorophenols as important intermediates in the chemical industry [7,8]. PCDEs can also be generated in the incineration of municipal waste [9,10]. Therefore, PCDEs have inevitably leaked into the environment and have been detected in water, sediment, soil, atmosphere and various biological samples at total concentrations of 0.351–1800 ng/L, 0–3,980,000 ng/g dry weight (dw), <38–6800 ng/g dw, 8.75 × 10^−3^–1.15 × 10^32^ pg/m^3^ and 0–50,924 ng/g lipid weight (lw), respectively (Table 1 and Table 2).

Since the negative log-transformed values of 298 K supercooled liquid vapor pressure (P_L_) of most PCDEs range from approximately 2 to 5, PCDEs may thus transport over long distances with the atmosphere [41]. For example, although there were no sources of pollution in the remote Arctic region, PCDEs were also detected in Arctic cod (*Arctogadus glacialis*) at concentrations of 2–21 ng/g lw [32,42]. Owing to the strong acid and alkali resistance and antioxidant capacity, PCDEs are persistent in various environmental matrices [1,17,18]. Moreover, the biological half-lives of tetra- to hepta-CDEs generally exceed 100 days in rainbow trout (*Salmo gairdneri*), which are almost equivalent to those of PCBs [43]. In addition, high lipophilicity renders PCDEs susceptible to accumulate in organisms and biomagnify through trophic transfer [44]. Toxicokinetic experiments showed that the absorption rate of PCDEs in fish were high at 2.4–48.9 μg/day, and the bioconcentration factor (BCF) could reach 1001–32,000 [44,45,46,47]. The bioaccumulation capacities of PCDEs are even higher than those of polychlorinated dibenzo-*p*-dioxins (PCDDs) and PCDFs in oligochaete worm (*Lumbriculus variegatus*) [48]. In some aquatic food chains, such as oligochaete worm (*Lumbriculus variegatus*) to white sucker (*Catostomus commersoni*), the biomagnification factors (BMFs) of PCDEs are 13.7 to 34.6 on a lipid-normalized basis, comparable to those of PCBs [11]. In addition, PCDEs have been detected in daily food and health products of humans [9,40].

**Figure 2:**
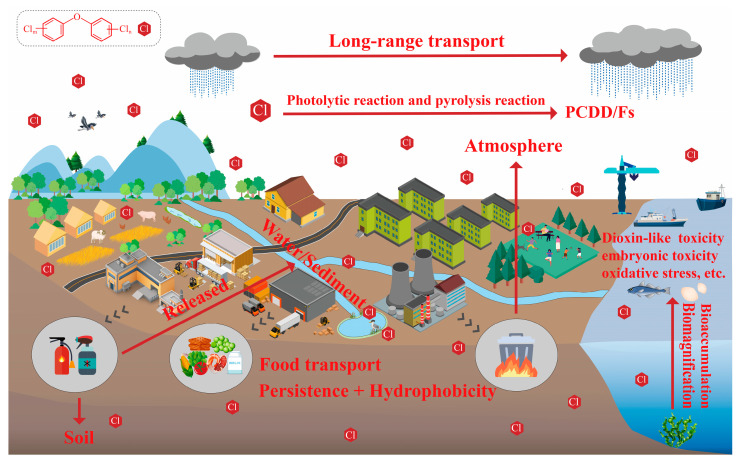
A schematic representation of the existing knowledge of PCDEs in terms of environmental toxicology and chemistry.

Toxicology studies have shown that the toxic effects of PCDEs in organisms are similar to dioxins. During the early life stages of fish, PCDEs may cause embryonic vascular hemorrhage, growth inhibition, deformity and death [49,50]. PCDEs can also cause oxidative stress in the liver of mice (*Mus musculus*) and disturb the balance of trace elements [51]. Exposure of mice (*Mus musculus*) to PCDEs during pregnancy resulted in reproductive developmental toxicity, such as reduced survival of fetuses and pups, and disturbed thyroid hormone secretion in maternal and fetal mice [52,53]. In addition, there is evidence that PCDEs may induce immunotoxicity through the mediation of the aryl hydrocarbon receptor (AHR) [54,55,56]. However, toxicological data of PCDEs are still limited probably due to the paucity of commercially available standards of PCDE congeners. It further leads to insufficient attention to the health and ecological risks brought by PCDEs, along with slowly increasing research on their toxic mechanisms, environmental exposure levels and environmental behavior. In this context, a systematic literature search was performed with a query based on the keywords of “Polychlorinated diphenyl ethers”, “PCDEs” and “polyhalogenated diphenyl ether”. PubMed, Web of Science and Google Scholar were used as search engines/databases. Publishing year was not restricted to retrieve as much of the available literature as possible. The relevant publications on PCDEs, focusing on the sources, environmental level, environmental behavior and fate, synthesis and analysis methods and toxicological research (Figure 2) were screened based on the examination of title, abstract and full text. The results and data were manually extracted and cross-checked by two authors. Additional relevant studies were identified from the reference lists of already identified publications. Finally, a total of 98 relevant publications were obtained from the literature search. Compared with reviews on PCDEs published previously [3,7], some new information and findings were summarized in this review: new sources including solid waste incineration [22], intermediates [6] and impurities in drugs, daily necessities and pesticides [57]; current environmental exposure levels [12,18,21]; main metabolism pathways in different aquatic organisms [58]; acute toxicity data for more species and relationships between it and structural parameters [59]; and relationships between bioaccumulation potentials and the number/location of substituting Cl atoms of PCDE congeners [58]. Furthermore, current research deficiencies were further proposed, and future research perspectives were explored to facilitate the environmental chemistry and toxicology research on PCDEs in the future.

## 2. Origin

### 2.1. Chlorophenol Preparations

Over the last several decades, there has been an accumulation of evidence that PCDEs primarily come from the production of chlorophenol preparations as by-products and impurities [8,9]. Commercial chlorophenols and their sodium and potassium salts were widely used as industrial wood preservatives, fungicides, insecticides, antifungal and antibacterial agents from the 1940s to 1980s [60]. At present, chlorophenols are still extensively used in chemical production as important intermediates, such as for the synthesis of clofibrate (CAS 637-07-0; a lipid-lowering drug), triclosan (CAS 3380-34-5; a broad-spectrum antimicrobial agent added in daily necessities, such as toothpaste), bifenox (CAS 42576-02-3; a nitrodiphenyl ether herbicide), 2-chlorophenyl N-methylcarbamate (CAS 3942-54-9; a carbamate insecticide) and triadimefon (CAS 43121-43-3; a triazole fungicide) [57]. Therefore, PCDEs may leak into the environment as impurities during the production, use, handling and disposal of chlorophenols and related products. It was found that the exposure levels of PCDEs in bearded gull (*Chlidonias hybrida*) eggs and night heron (*Nycticorax nycticorax*) eggs collected from the Yangtze River Delta were related to the use and discharge of pentachlorophenol and sodium pentachlorophenate [26]. In commercial chlorophenols and related products, the content of PCDEs ranges from 4.4 to 1000 mg/kg wet weight (ww) (Table 3), which is related to the synthesis processes and the ratios of reactants. Moreover, the annual production of global chlorophenols was estimated to be 200,000 tons [61,62]. Thus, according to the estimated annual production of chlorophenols and the average content of PCDEs in them, at least 66 tons of PCDEs per year have been produced as impurities in chlorophenols globally.

### 2.2. Municipal Waste Incineration

Municipal waste incineration is another important potential source of PCDEs. The concentration of PCDEs in the stack flue gas of some electric arc furnaces are 0.0115 ng/Nm^3^ [22]. They were detected at concentrations of 1.48–10.3 ng/Nm^3^ in the flue gas of a small household waste incinerator, but the level of PCDEs dramatically increased with the addition of chlorine-containing plastic and Cu, reaching 279,000 ng/Nm^3^ [64]. Seventy-nine PCDE congeners were analyzed in fly ash samples from a municipal waste incineration plant in Germany. The total concentration of all PCDE congeners was 93 µg/kg fly ash, and each PCDE congener had the same level at µg/kg fly ash [9]. In fly ash samples of Finnish municipal waste incineration plants, total PCDE concentrations were detected at 0.1–3.8 µg/kg fly ash [30]. Moreover, chlorinated compounds, such as chlorobenzene, chlorophenol and chlorophenoxy, can condense to PCDEs in the early stages of municipal waste combustion when the combustion chamber temperature is lower than 450 °C, compared to the complete combustion stage when the combustion temperature reaches over 800 °C [10,65]. Unlike the highly chlorinated PCDEs in chlorophenol formulations, the number of substituted Cl atoms of PCDEs from municipal waste combustion is mainly between two and six [9]. This difference may be attributed to the composition of the incineration material. One study suggested that low-chlorine PCDEs were the main congeners in the flue gas of a small household waste incinerator without chlorine-containing plastic, while the percentage of high-chlorine congeners increased by the co-presence of chlorine-containing plastics or Cu [64]. The presence of FeCl_3_ and CuCl_2_ in solid waste also increases the formation of highly chlorinated PCDEs on a simulated fly ash surface, whereas Fe_2_O_3_ and CuO increase the formation of lower chlorinated PCDEs [66,67]. Furthermore, without oxygen, Fe_2_O_3_ catalyzes the formation of PCDEs, whereas CuO reduces PCDE’s formation [67].

## 3. Physicochemical Properties, Environmental Levels, Behavior, Fate and Degradation

### 3.1. Physicochemical Properties

The physicochemical properties related to the environment behavior and fate of pollutants have been determined for 106 PCDE congeners by direct chromatographic methods (Table S2) [41,68]. However, given the time- and cost-consuming characteristics to evaluate the physicochemical properties experimentally as well as unavailability of standards for the remaining 103 PCDE congeners, various quantitative structure–property relationship (QSPR) methods have been developed and applied to predict the physicochemical properties based on diverse molecular structural descriptors and regression models. For example, seventeen theoretical molecular structural descriptors and partial least squares (PLS) regression were used to predict the P_L_ and n-octanol/water partition coefficient (K_OW_) of 209 PCDE congeners [69]. Linear relationships were established between gas-chromatographic relative retention time (RRT), K_OW_, PL and aqueous solubility (S_W,L_) of PCDEs and some structural descriptors derived from molecular surface electrostatic potentials by a multiple linear regression (MLR) method and used to predict the physicochemical properties of PCDE congeners not determined experimentally [70]. QSPR models were developed by molecular electronegativity distance vector (MEDV-4) and MLR methods to estimate the P_L_, K_OW_ and S_W,L_ of 209 PCDE congeners [71]. Based on the number of substituting Cl atoms on the different positions of parent compound diphenyl ether and the number of relative positions for these Cl atoms, a QSPR model was established by the theoretical linear solvation energy relationship (TLSER) method to predict the P_L_ of PCDEs with correlation coefficients R^2^ of 0.991 [72]. An MLR approach was utilized to develop QSPR models to predict the P_L_ of 106 PCDEs based on calculated molecular descriptors [73]. The S_W,L_ values of five PCDE congeners were predicted using a PLS method [74]. The physicochemical properties predicted from the QSPR models mentioned above are listed in Table S3 [69,71,72,73,74]. The experimental and predicted results show that logP_L_, logK_ow_ and logS_w,l_ of PCDEs range from −5.97 to −0.27, 4.38 to 8.31 and −12.95 to −4.21, respectively. These physicochemical properties indicate that PCDEs tend to accumulate in environments rich in organic matter, such as soils, sediments and organisms.

### 3.2. Environmental Levels

#### 3.2.1. Water

To the best of our knowledge, only three studies are available on the levels of PCDEs in water. Samples from the contaminated area of Whitby Harbor and a bridge near the entrance to Pringle Creek on the north shore of Lake Ontario were analyzed; 45 PCDE congeners were found in the semi-permeable membrane device (SPMD) at total concentrations of 0.68–7.07 ng/L [11]. In China, 15 PCDE congeners were detected in surface water samples from the Nanjing section of the Yangtze River [13]. The total concentration ranged from 1150 to 1800 ng/L and 730 to 1300 ng/L during the low- and high-water periods, respectively, with CDE 30 being the dominant congener. In the next study by the same group, the total concentrations of the PCDE congeners ranged from 0.351 to 2.021 ng/L in surface water samples from Chaohu Lake and its eight main tributaries in China, with CDE 30 (20.63%), CDE 28 (9.78%) and CDE 37 (9.52%) as the major congeners [12]. In general, PCDEs with less substituted Cl atoms have lower logK_ow_ and relatively higher water solubility [41]. Therefore, lower chlorinated PCDEs, such as mono-, di- and tri-CDEs are more easily transferred to the aqueous phase than higher chlorinated congeners [12]. The presence of PCDEs in water may be associated with surrounding or upstream industrial production and human activities, such as the production and use of chlorophenols, clofibrate, triclosan, bifenox, 2-chlorophenyl N-methylcarbamate and triadimefon [11,12,13,57]. Studies showed that CDE 37 and 77 could induce severe oxidative damage in green algae (*Scenedesmus obliquus*), water flea (*Daphnia magna*), zebrafish (*Danio rerio*) and crucian carp (*Carassius auratus*) at environmentally relevant concentrations [59,75].

#### 3.2.2. Sediment and Suspended Particulate Matter

PCDEs tend to accumulate in the sediment compared to water due to their higher hydrophobicity. The pollution of sediment by PCDEs was first reported for Whitby Harbour on the north shore of Lake Ontario in 1981 [76]. Subsequently, the environmental exposure of PCDEs has gradually received attention. The mean concentrations of total PCDE congeners in sediments of the contaminated area of Whitby Harbour were between 622 and 1929 ng/g dw in 1995 [11]. The average detection concentration of PCDEs in Lake Ontario was 1.30 ng/g dw, which was comparable to that of PCDDs (1.10 ng/g dw) and PCDFs (2.44 ng/g dw) [18]. In the sediment of Kymijoki River in Finland, which was highly contaminated by PCDEs due to the intensive production and use activities nearby of chlorophenol in the 19th century, the total concentration of PCDEs was determined in the range of approximately 130 to 554 ng/g dw (50 congeners tested) in 1993 [15], 8.79 to 606 ng/g dw (40 congeners tested), except for the reference sediment, in 1997 [16], and 85 ng/g dw (nine congeners tested) in 2001 [17]. The types and quantities of the measured compounds were different; therefore, it is difficult to judge the changing trend of PCDEs concentration in sediments of the Kymijoki River year by year. In industrially developed areas of eastern China, sediment samples were collected from Chaohu Lake and the Nanjing section of the Yangtze River, where the total concentrations of 15 PCDE congeners were in the range of 0.279–2.47 ng/g dw and 1.24–3.98 ng/g dw, respectively [12,13]. The level of PCDEs (mean: 1.30 ng/g dw) in the sediments of Chaohu Lake were higher than that of structurally similar polybrominated diphenyl ethers (PBDEs) tested (mean: 0.714 ng/g dw) [77], while lower than that of PCBs (mean: 12.07 ng/g dw) [78]. In addition to sediments, PCDEs in suspended particulate matter (SPM) of Chaohu Lake were also detected. The result showed that the mean total concentration of PCDEs in SPM was comparable to that in the sediment, which was 1.15 ng/g dw, lower than that of PBDEs (mean: 232.5 ng/g dw). In the SPM of the upper Narragansett Bay, the detected concentrations of tri-CDEs and tetra-CDEs were 0.03 ppt dw and 0.06 ppt dw, respectively, which were lower than that of tri-CDF (0.25 ppt dw) [19]. Furthermore, compared with the chlorinated degree of PCDEs in water, PCDEs with more chlorine atoms were more likely to accumulate in sediment and SPM.

#### 3.2.3. Soils

By contrast, very little information is available on the levels of PCDEs in soils. An earlier study showed that the total concentration of 19 PCDE congeners ranged from <38 to 6800 ng/g dw in soils at 5 contaminated sawmill sites in Sweden [20].

#### 3.2.4. Atmosphere

Only one report to our knowledge has recently showed the levels of PCDEs in the atmosphere. That is, the atmospheric occurrence of six PCDE congeners were investigated over the rural area and the Pacific Ocean near Taiwan and the northern Philippines [21]. An elevated mean level of PCDEs was found in the ambient air of the rural area (0.014 pg/m^3^) compared with that found in the oceanic atmosphere (0.00875 pg/m^3^). CDE 28 was the predominant congener, accounting for 98.3 and 95.8% of the total PCDEs in the oceanic atmosphere and the ambient air over the land, respectively.

#### 3.2.5. Biological Organisms

Organisms are susceptible to contamination by PCDEs in the environment due to their lipophilic nature. The presence of PCDEs in organisms was first identified in marine organisms, including clam (*Mercenaria mercenaria*), mussel (*Mytilus edulis*) and lobster (*Honarus americanus*), from Narragansett Bay in the United States [19]. PCDEs were also detected in freshwater fish in the North American Great Lakes. The total concentration of 28 monitored PCDE congeners ranged from 24 to 891 ng/g lw in lake trout (*Sulvelinus namaycush*) and walleye (*Stizostedion vitreum vitreum*) collected from the Great Lakes on a whole-fish basis [29]. Penta-, hexa- and hepta-chlorinated congeners were the most abundant homologue groups, representing approximately 80 to 90% of the total concentrations. In another study, the occurrence of 15 PCDE congeners was examined in whole fish samples of common carp (*Cyprinus carpio*) and northern pike (*Esox lucius*) caught from Whitby Harbour on the north shore of Lake Ontario [28]. The total levels of PCDEs varied from 768 to 14,005 ng/g ww, well above the detected concentrations of PCDFs (58–254 pg/g ww). In a later investigation on 8 fish species, including common shiner (*Notropis cornutus*), rosyface shiners (*Notropis rubellus*), spottail shiner (*Notropis hudsonius*), pumpkinseed (*Lepomis gibbosus*), yellow perch (*Perca flavescens*), brown bullhead (*Ameiurus nebulosus*), white sucker (*Catostomus commersoni*) and northern pike (*Esox lucius*), collected also from Whitby Harbour, the total lipid-normalized concentrations of 45 PCDE congeners in muscle samples for each species ranged from 100 to 2857 ng/g, 23231 to 43,231 ng/g, 20,706 to 96,529 ng/g, 30,417 to 68,250 ng/g, 4200 to 130,333 ng/g, 7538 to 213,231 ng/g, 16,714 to 174,571 and 21,000 to 47,000 ng/g, respectively [11]. CDE 99, 153 and 154 were the dominant congeners, and CDE 47, 74, 100, 118, 163, 182 and 184 were also significant. In addition to fish in inland lakes and coastal waters, PCDEs were also indirectly detected in deep sea fish through investigating levels of 106 PCDE congeners in 2 cod liver oils made from North Atlantic deep sea fish [9]. The total PCDE levels were 49 and 659 ng/g lw, respectively. These studies reflect the common presence of PCDEs in organisms in both marine and freshwater environments.

PCDEs have also been detected in organisms in other countries and regions. In oligochaete worm (*Lumbriculus variegatus*), chironomids and northern pike (*Esox lucius*) collected from sampling sites in the Kymijoki River in Finland, located downstream of an adjacent Ky-5 (which was a chlorophenol wood preservative) production plant, the total concentrations of 40 or 50 PCDE congeners were detected ranging from 215 to 1325 ng/g lw, 0 to 1200 ng/g lw and 677 to 706 ng/g lw, respectively [15,16]. The patterns of PCDE levels in these organisms were similar and resembled that in the sediments collected at the same sampling sites, and these dominant congeners were also abundant in Ky-5 as well. The major PCDE congeners detected in salmon from the Tenojoki Rive, Lake Saimaa and the Simojoki River in Finland were also similar and abundant in Ky-5 too [30]. Moreover, the congener patterns appear to be similar to those detected in Whitby Harbour fish [11]. It indicates that PCDEs contamination in the two regions may be attributed to the production or use of Ky-5 there.

**Figure 3:**
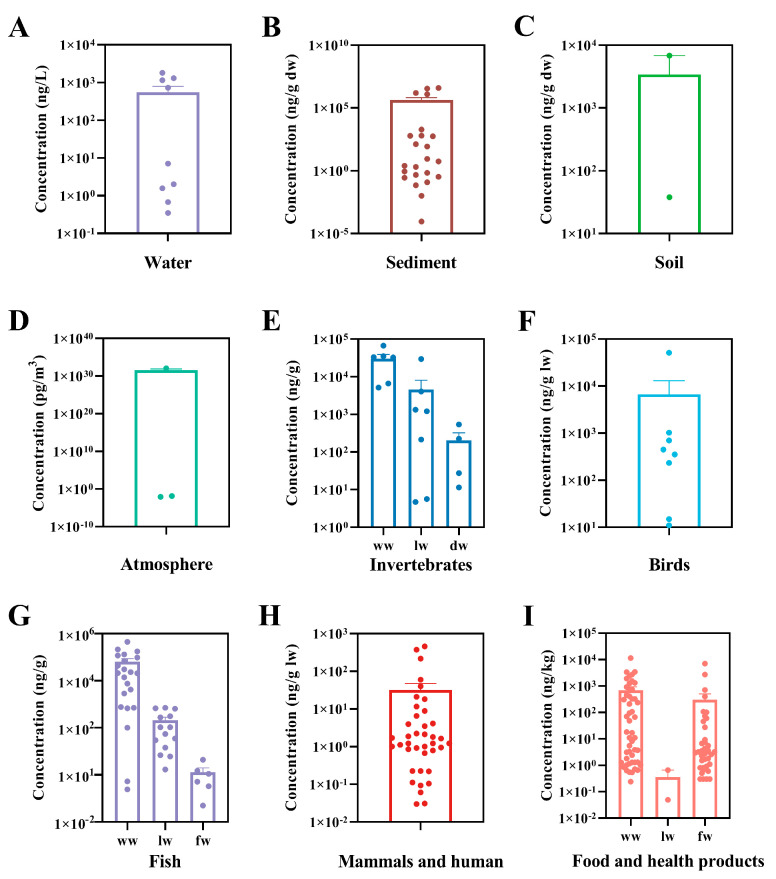
Concentrations of PCDEs detected in water (**A**), sediment (**B**), soil (**C**), atmosphere (**D**), invertebrates (**E**), birds (**F**), fish (**G**), mammals and human (**H**) and food and health products (**I**).

PCDEs in organisms are acquired not only by bioconcentration from the ambient environment, but also by biomagnification throughout the food chain. PCDEs have been detected in birds and mammals that eat fish and other aquatic organisms. For example, eggs of fish-eating birds, including common tern (*Sterna hirundo*), black skimmer (*Rynchops niger*) and bald eagle (*Haliaeetus leucocephalus*) from Rhode Island, Louisiana, Michigan and Ohio were examined, and it was found that the total concentrations of three PCDE congeners tested ranged from 11 to 900 ng/g ww [24]. The high concentrations of PCDEs (sum of 7 congeners) were also found in eggs of black-crowned night herons (*Nycticorax nycticorax*) from Tianmu Lake and whiskered terns (*Chlidonias hybrid*) from East Tai Lake in China with levels ranging from 11 to 450 ng/g lw and 15 to 700 ng/g lw, respectively [26]. They were well above the detected total concentrations of PCDD/Fs of 0.38–19 and 2.6–33 ng/g lw in the two birds, respectively. In an investigation within the Baltic Sea area as the most polluted brackish water area in the world, the concentrations of individual PCDE congeners were detected ranging from <3 to 79 ng/g lw in eggs of black guillemots (*Cepphus grylle* L.) and from <5 to 13,000 ng/g lw in breast muscle of white-tailed sea eagles (*Hallaeetus albicilla* L.) as a top predator of the Baltic food chain [25]. The total concentrations of the 50 tested PCDE congeners varied from 233 to 354 ng/g lw and 1027 to 50,924 ng/g lw, respectively. They were also significantly higher than those of PCDD/Fs, i.e., 3.9–4.0 ng/g lw in black guillemots (*Cepphus grylle* L.) and 1.6–133 ng/g lw in white-tailed sea eagles (*Hallaeetus albicilla* L.). In mammals, such as seals, high levels of PCDEs were also detected. The contents of 50 individual congeners ranged from <0.3 to 62 ng/g lw in blubber of ringed seals (*Phoca hispida botnica*) and grey seals (*Halichoerus grypus*) from the Gulf of Finland in the Baltic Sea with the total concentrations of 39.9–373.9 ng/g lw [14]. PCDEs were at similar levels in the seal blubber compared to fish captured here and from the Kymijoki River that finally flows into the Gulf of Finland [16,27]. In blubber samples of a Baikal seal (*Phoca sibirica*) from Lake Baikal in East Siberia of Russia and several ringed seals (*Phoca hispida saimensis*) from Lake Saimaa in Southeast Finland, the total concentrations of the 50 congeners were found to be 60 ng/g lw and 217–459 ng/g lw, respectively [15]. In blubber samples of harbor seals (*Phoca vitulina*) captured from the Salish Sea in north–western North America, lower total contents of PCDE congeners (6.5–21 ng/g lw; sum of 46 congeners) were measured, which might be due to light PCDEs contamination in North America [33]. Furthermore, studies have demonstrated the presence of PCDEs in human adipose tissue. Tetra- to deca-CDE congeners in human adipose tissue collected from Canadian municipalities were analyzed. CDE 206 and 209 were found to be in the range of 0.1–2.9 ng/g lw, and the mean level of CDE 206 in males was greater than that in females [34]. Six hexa- to deca-CDE congeners were also detected in human adipose tissue from the USA, where the predominant congener was CDE 206 with concentrations ranging from 0.6 to 1.4 ng/g lw [35]. In addition, it was reported that the concentrations of 50 individual PCDE congeners varied between <0.5 and 7.9 ng/g lw in Finnish human adipose tissue, which were comparable to the levels of PCDD and PCDF congeners (<5 to 7700 pg/g lw) [35]. The main origin of PCDEs found in humans may be contaminated food. Human exposure to PCDEs through the diet was first reported in Catalonia (Spain) in 2004 [36]. PCDEs were detected in a number of foodstuffs available in the local market. The total PCDE concentrations in fresh hake (*Rexea solandri*), fresh sardine (*Sardina pilchardus*), mussels and tinned fish were 45.9–707, 400–2707, 59.8–107 and 3.3–71.9 pg/g ww, respectively. Total dietary intake of PCDEs through fish and shellfish was estimated to be 38 ng/day by a standard male adult of 70 kg body weight and aged between 20 and 65 years in Catalonia (Spain), which was slightly higher than PBDEs of approximately 31 ng/day. Moreover, PCDE intake was always higher in males than in females for people under 45 years old due to a greater food intake by males. In a subsequent study by the same research group, the concentrations of PCDEs were determined in 14 edible marine species widely consumed by the population of Catalonia (Spain) [38]. The highest PCDE levels (pg/g ww) were found in red mullet (*Mullus barbatus*; 7088) followed by sardine (*Sardina pilchardus*; 1829), anchovy (1606), tuna (*Scombridae gen.* sp.; 1292) and mackerel (1031). Children aged 4–9 years (boys 0.88 ng/kg/day and girls 0.73 ng/kg/day) showed the highest PCDE intake when judged by the average body weight [79]. Dietary intake of PCDEs in athletes was also evaluated [37]. In general, sportsmen and sportswomen showed a lower daily dietary intake than the general population due to ingesting lower amounts of fish and seafood. In another survey of PCDEs in foodstuffs in Catalonia (Spain) in 2006, the dietary intake of PCDEs was 51.68 ng/day for a standard male adult of 70 kg body weight, increasing by 26% compared to the previous survey (41 ng/day) in 2000, with fish and seafood being the main contributors to this increase [36,39]. In addition, the influence of different cooking processes including frying, grilling, roasting and boiling on the levels of PCDEs in various foodstuffs was evaluated. Studies showed that almost all cooking processes enhanced the total PCDEs levels in fish and meat samples [40,80]. Detailed information about the levels of PCDEs in various environmental media and biota reported previously is provided in Figure 3 and Table 1 and Table 2, respectively.

### 3.3. Environmental Behavior and Fate

#### 3.3.1. Bioaccumulation and Biomagnification

The logK_ow_ values of all PCDE congeners were greater than four (Table S2) [41]. Thus, PCDEs are superlipophilic and tend to accumulate in organisms and biomagnify through the food chain [44]. The accumulation rate and extent of PCDEs in fish are generally similar to those of PCBs, and some PCDE congeners are highly bioaccumulative with a logBCF > 3.70 L/kg [44,46]. The bioaccumulation of PCDEs was revealed to be more intense than that of PCDD/Fs in benthic oligochaete worm (*Lumbriculus variegatus*) following 28-day exposure to contaminated sediments [48]. The logBCF of CDE 47 was measured to be 4.09 in rainbow trout (*Salmo gairdneri Richardson*) muscle [47]. Recently, the bioaccumulation of 12 PCDE congeners was studied in 3 model aquatic organisms, including green algae (*Scenedesmus obliquus*), water fleas (*Daphnia magna*) and zebrafish (*Danio rerio*) [58]. The logBCF values were found to be in the range of 2.94–3.77, 3.29–4.03 and 2.42–2.89 L/kg ww, respectively. Moreover, the logBCF values increase with the increasing number of substituted Cl atoms, with the exception of CDE 209 maybe due to its large molecular volume preventing it from penetrating through the cell membrane. In addition, similar to PCBs, the number of Cl atoms at *para*- and *meta*-position may be another major positive contributing factor for BCFs in the case of the same number of substituted Cl [58].

Some PCDE congeners exhibited biomagnification potentials comparable to some PCDD/Fs, PCBs and PBDEs. For example, as mentioned above, the total concentration of PCDEs detected in breast muscle of white-tailed sea eagles (*Hallaeetus albicilla* L.), as a top predator of the Baltic food chain, was 1027 to 50,924 ng/g lw, which was significantly higher than that tested in eggs of black guillemots (*Cepphus grylle* L.) at a lower trophic level ranging from 233 to 354 ng/g lw [25]. Moreover, they were significantly higher than the total concentrations of PCDD/Fs, i.e., 3.9–4.0 and 1.6–133 ng/g lw in black guillemots (*Cepphus grylle* L.) and white-tailed sea eagles (*Hallaeetus albicilla* L.), respectively. In a benthic food chain from chironomids to white sucker (*Catostomus commersoni*) and a pelagic food chain from plankton to pumpkinseed (*Lepomis gibbosus*) in Whitby Harbour of Lake Ontario, the BMFs (on a ww basis) of six PCDE congeners, including CDE 47, 74, 99, 100, 153 and 154 ranged from 1.4 to 2.3 and 2.7 to 4.7, respectively [11]. The BMFs (on a ww basis) for PCBs were calculated in a food chain from mysids (*Mysis relicta*) to alewife (*Alosa pseudoharengus*) in Whitby Harbour in another study [81]. They were 3.5 and 4.5 for PCB 74 and PCB 99, respectively, comparable to those for corresponding PCDE congeners in the pelagic food chain (i.e., 4.3 and 4.0 for CDE 74 and CDE 99, respectively) [11]. In addition, in a simulated aquatic food chain from green algae (*Scenedesmus obliquus*) to zebrafish (*Danio rerio*) through water fleas (*Daphnia magna*), the BMFs for 12 PCDE congeners varied from 0.800 to 3.31 (on a ww basis) or 0.881 to 3.64 (lipid-normalized) [58]. They were also comparable to the lipid-normalized BMFs of 18 PBDEs, which were determined in a highly contaminated freshwater food chain from South China ranging from 0.26 to 4.47 [82]. Furthermore, there is evidence that the BMFs of PCDEs may increase with increasing number of substituted Cl atoms [11,58].

#### 3.3.2. Tissue Distribution, Metabolism and Excretion

Biological half-lives of tri- to deca-CDEs in rainbow trout (*Salmo gairdneri*) reached 46 to >300 days, which are almost equivalent to those of PCBs [43]. Moreover, their mean half-life values tend to increase with chlorine content except for CDE 209 with a half-life of 46 days. After being absorbed by brook trout (*Salvelinus fontinalis*), PCDEs initially enter the blood and liver and then redistribute to adipose tissue and muscle [45]. A study on the tissue distribution of CDE 99 in rats (*Rattus norvegicus*) showed that the highest concentration was observed in fat, followed by skin, liver, kidney and muscle, and the concentrations declined almost to the background levels in most tissues except for fat on day 21 [83]. Excretion studies of CDE 99 in rats administered at a single oral dose of 10 mg/kg showed that approximately 55% and 1.3% of the oral CDE 99 were excreted in feces and urine, respectively, in 7 days [83]. Thus, fecal excretion may be the main disposition pathway for PCDEs.

Studies showed that PCDEs can be metabolized via dechlorination, scission of the ether bond and hydroxylation and methoxylation of aryl nuclei in organisms. It was found that aromatic hydroxylation was the main metabolism pathway in rats (*Rattus norvegicus*), and it tended to take place *ortho* and *meta* to the ether bond [83,84]. Additionally, if PCDEs contain at least one Cl atom at the *ortho* position of benzene rings relative to the ether bond, predioxins may form via *ortho*-hydroxylation [84]. Moreover, lower chlorinated PCDEs seem to be metabolized more rapidly than higher chlorinated congeners [45]. The metabolic pathways of CDE 15 were investigated in three aquatic organisms, including green algae (*Scenedesmus obliquus*), water fleas (*Daphnia magna*) and zebrafish (*Danio rerio*) [58]. In green algae and water fleas, dechlorination was found to be the predominant metabolic mode, while methoxylation was the dominant metabolism pathway in the liver of zebrafish, followed by dechlorination and hydroxylation, which was in contrast to the finding by Tulp et al. in rats (*Rattus norvegicus*) [84].

#### 3.3.3. Long-Range Transport

The partitioning of organic pollutants between gaseous and aerosol or particulate phases is related to the physicochemical property P_L_. Thus, the P_L_ of organic substances can be used to estimate their distribution, transport and fate in the environment. Pollutants with P_L_ < 10^−5^ Pa are almost entirely adsorbed on the solid airborne particles, while they preferentially distribute into the gas phase when P_L_ is between 10^−5^ and <10^−2^ Pa [85]. The –log P_L_ values of PCDEs range from 0.27 for CDE 1 to 5.80 for CDE 209, increasing with increasing chlorination degree (Table S2) [41]. The range of –log P_L_ values of PCDE are in the same order of magnitude as PCBs (0.56 for PCB 3 to 4.66 for PCB 208) [86]. The –log P_L_ values of tetra- to nona-CDE congeners range from approximately 2 to 5 (Table S2) [41]. It indicates that most PCDE congeners may transport over long distance with the atmosphere in gaseous phase and atmospheric particulate phase [41].

### 3.4. Photolysis and Pyrolysis Reactions

PCDEs have been shown to undergo natural photolysis under environmental conditions and pyrolysis to generate PCDD/Fs, hydroxylated PCDEs (HO-PCDE) and chlorobenzene [87,88]. Among them, PCDD/Fs, especially PCDFs, are the main decomposition products [87,89]. The photolysis pathways of PCDEs include photodechlorination, C-O bond photodissociation and photocyclization to form PCDFs [88]. The photoactivity of PCDEs increases with the increasing chlorination degree [90]. When the pyrolysis temperature is higher than 700 °C, PCDEs are almost completely decomposed [91]. The highest yield of PCDFs is reached at 600 °C [91]. The pyrolysis of PCDEs also has three routes including C-O bond dissociation, ring-closure reaction to form PCDFs, and the addition of a ground state oxygen molecule at an apparent *ortho* radical site to form PCDDs [89,91]. The formation of PCDD/Fs from PCDEs needs at least one *ortho*-chlorine for both photolysis and pyrolysis reactions, during which *ortho*-H_2_, *ortho*-HCl and/or *ortho*-Cl_2_ are lost [89,91,92]. The degree and pattern of chlorination did not affect the formation pathways of PCDD/Fs [80]. In addition, this cyclisation reaction can be promoted by palladium (II) acetate [93,94]. Therefore, PCDEs should also be noteworthy as an important source of precursors for the formation of PCDD/Fs in the environment.

## 4. Synthetic and Analytical Methods

Studies have shown that the coupling reaction of chlorinated diphenyliodonium salt with chlorinated phenols is the common route to synthesize most individual PCDE congeners [95]. However, some iodinated side-products can be formed, such as chlorinated iododiphenyl ethers, which cannot be separated from desired products [95,96]. Moreover, this approach is time-consuming due to inefficient preparation procedure of diphenyliodinium salts. A cuprous iodide-catalyzed Ullman coupling reaction was used later to synthesize ten PCDE congeners with chlorinated iodobenzene and chlorinated phenol in alkaline conditions [13,97]. The advantages of this synthetic route are easy operation and less side-products. The disadvantage is that chlorinated phenols with Cl atom(s) at the *ortho* position of the hydroxyl group cannot be used as reactants due to the formation of PCDD/Fs as by-products. Mono- and di-CDEs can also be synthesized using copper diacetate-catalyzed Chan–Lam coupling reactions of aryl boronic acids and phenols [98,99]. However, the yield of CDE 4 is low (only 8%) due to the sterically hindered 2-chlorophenylboronic acid.

Although gas chromatography combined with mass spectrometry (GC–MS) is the common method for the determination of PCDEs in samples [11,12], high resolution gas chromatography combined with high resolution mass spectrometry (HRGC–HRMS) is preferred due to higher sensitivity and specificity [16,20]. However, several procedures are required before analysis of PCDEs by GC–MS or HRGC–HRMS, depending on the types of environmental samples. For water samples, the main procedures include filtration through 0.45 μm membrane, solid phase extraction and cleanup [12,13]. For solid environmental samples, such as sediment and biota samples, they mainly involve sample pretreatment (e.g., drying, homogenization and acid/base digestion), solvent extraction and cleanup [15,16]. Samples are commonly dried by freeze-drying [11] or sodium sulfate [9]. The dried samples are homogenized using a homogenizer [100] and acid or base digestion [30]. Extraction of PCDEs from water and milk samples have been performed via solid phase extraction [12,13] and Soxhlet extraction [36], respectively, while for solid samples, Soxhlet extraction [16], ultrasonic extraction [15], column extraction [101] and accelerated solvent extraction [32] have been used. Cleanup techniques mainly include bulk matrix removal and adsorption chromatography for removing interference to the subsequent analysis. Bulk matrix removal can be performed by acid treatment [14] or liquid–solid chromatography on Florisil [31], silica gel [19], modified silica gel [40] and modified celite [101] to remove material in biota and sediment extracts, such as lipids, which can disturb final analysis. Adsorption chromatography is used to separate out PCDEs from other compounds, such as PCBs, PCDDs and PCDFs. Column chromatographies on Florisil [16], silica gel [13], alumina [102] and carbon fiber [103] have been successfully used to remove impurities.

## 5. Toxicology Research

Despite the concerns about PCDEs as persistent, bioaccumulative and potential toxic substances in the environment, limited studies have been carried out on their toxic effects. Current studies show that PCDEs can cause various adverse effects including lethal toxicity [45,49,50], growth inhibition [49,104], tissue damage [50,105,106,107], reproductive toxicity [52,53], developmental toxicity [49,105,106], immunotoxicity [54], oxidative stress [51,75,104,108,109] and endocrine disorder [53] in mammals, fish and/or plankton (Table S4) [45,49,50,51,52,53,54,75,104,105,106,107,108,109,110]. Subchronic toxicities of several di- to tetra-*ortho*-chlorinated congeners have been demonstrated to be moderately toxic in rats (*Rattus norvegicus*), including CDE 99, 100, 132, 139, 153 and 184 [105,106]. Growth inhibition and thyroid gland injury were also detected in these studies [105,106]. In addition, liver injuries were found in CDE 15-exposed mice (*Mus musculus*) [51]. Hepatic oxidative stress was observed in CDE 37-exposed crucian carp (*Carassius auratus*) at environmentally relevant concentrations [75]. Subchronic exposure to CDE 3, 15, 37, 77 and 118 caused oxidative stress in the liver and ovary of adult zebrafish (*Danio rerio*), and CDE 15 could also induce liver nuclei enlargement, necrosis, hepatocyte vacuolation and the developmental inhibition of ovarian cells [107]. Moderate and high acute toxicities, including lethal toxicity or growth inhibition, were observed for CDE 3, 7, 15, 28, 30, 37, 66, 77, 99, 118 and 209 in zebrafish (*Danio rerio*), water fleas (*Daphnia magna*) and green algae (*Scenedesmus obliquus*) [59,104], as well as for CDE 3, 7, 28 and 74 in brook trout (*Salvelinus fontinalis*) [45], which were generally comparable with those of certain PCBs and PBDEs [59]. Moreover, CDE 77 could also induce oxidative damages in zebrafish (*Danio rerio*), water fleas (*Daphnia magna*) and green algae (*Scenedesmus obliquus*) at environmentally relevant concentrations [59]. The substitution number and pattern of chlorine atoms seem to influence the acute toxicities of the congeners tested, i.e., the non-*ortho*-substituted may have higher acute toxicities than the *ortho*-substituted [59]. QSPR models indicate that their acute toxicities may be correlated with their molecular polarizability (α) and the energy of the lowest unoccupied molecular orbital (E_LUMO_) [59]. The LC_50_ or EC_50_ values of PCDE congeners based on acute toxicities from previous studies are listed in Table S5 [45,49,50,59,75].

Reproductive and developmental toxicities were found in PCDEs-administered mice (*Mus musculus*). For example, the number of pups born per female mated and the number of pups surviving per litter born were both decreased following exposure of parent female mice (*Mus musculus*) to CDE 71 and 154 from the 6th to 15th day after pregnancy, while CDE 102 and 153 decreased the number of litters born per female mated, without decreasing postnatal survival [52]. Maternal exposure to CDE 71, 102 and 153 also depressed thyroxine levels in both sexes of juvenile rats (*Rattus norvegicus*) [53]. Moreover, CDE 71 and 102 caused hypothyroidism in pregnant rats (*Rattus norvegicus*) as well. It was also found that 28-day subchronic exposure of adult rats (*Rattus norvegicus*) to CDE 99, 100, 132, 139, 153 and 184 caused thyroid damage [105,106]. These findings showed the thyroid-related endocrine-disrupting activity of PCDEs. In addition, developmental toxicities were observed in early life stages of CDE 15-exposed zebrafish (*Danio rerio*) at 10 mg/L, such as delayed hatching, growth inhibition and malformations, including pericardial edema, yolk sac edema, spine deformation and tail curvature [49]. CDE 15 can also cause the developmental inhibition of ovarian cells in adult female zebrafish (*Danio rerio*) perhaps due to mitochondrial disappearance [107]. Moreover, CDE 3, 15, 37, 77 and 118 can significantly enhance mRNA expression of the *vtg1* gene in the liver of adult male zebrafish (*Danio rerio*), among which CDE 3 and 15 can also increase vitellogenin content in blood samples, suggesting that some PCDE congeners may be estrogen endocrine disruptors [107].

Among the PCDE congeners tested previously for immunotoxicity, most were found to be immunosuppressive in rodents, including CDE 77, 101, 118, 126, 153, 154, 156, 184, 206, 207, 208 and 209, except for CDE 167, although they were >200 times less immunotoxic than 2,3,7,8-tetrachlorodibenzo-*p*-dioxin (2,3,7,8-TCDD) [54,55,106]. For the PCDE congeners, increasing *ortho*-substitution is less effective in reducing the toxicity of these congeners compared to the well-recognized *ortho* effects reported for the PCBs [56]. This may be because the ether bridge increased bond length between two phenyl rings, thereby diminishing the effects of *ortho* substituents on the toxic potencies. A quantitative structure-activity relationship (QSAR) model was developed for PCDEs based on the immunotoxicity values and electronic properties of the 12 PCDE congeners [111]. It showed that congeners with substitutions at positions three and four tended to have higher immunotoxicity and a lower frontier orbital energy gap. However, higher exposure doses (≥25 μmol/kg) were needed to induce immunosuppressive effects for CDE 206, 207, 208 and 209 in less AHR-responsive DBA/2 mice (*Mus musculus*) compared with the doses of 2.5 to 10 μmol/kg in AHR-responsive C57BL/6 mice (*Mus musculus*) [54]. Moreover, for CDE 77, 101, 118, 126, 153 and 156, an excellent linear correlation was observed between their immunotoxicity and induced ethoxyresorufin-*O*-deethylase (EROD) or aryl hydrocarbon hydroxylase (AHH) activity as markers of AHR activation in mice (*Mus musculus*) [56]. These findings indicate that the immunotoxicity of PCDEs may be mediated by AHR. In addition, it was found that CDE 28, 74, 77, 126, 128, 105, 156, 170, 177, 180, 187, 195, 203 and 209 increased liver monooxygenase activities and/or cytochrome *P*-450 levels in rats (*Rattus norvegicus*) [110,112]. Significantly increased induction of EROD activity was also observed in CDE 77-exposed rainbow trout (*Oncorhynchus mykiss*) by gavage intubation and CDE 74-exposed speckled trout (*Salvelinus fontinalis*) by intraperitoneal injection [109,112]. Therefore, some PCDE congeners may be potential dioxin-like compounds.

Based on the immunotoxicity and AHR-related enzyme activity of the above PCDEs, an interim toxicity equivalence factor (TEF) value of 0.001 relative to 2,3,7,8-TCDD was proposed for *non*- and mono-*ortho*-PCDEs in mice (*Mus musculus*) [55]. TEF values for CDE 77, 118 and 105 in Japanese medaka (*Oryzias latipes*) were also estimated based on acute mortality data, i.e., 0.00003, 0.00001 and 0.00056, respectively [50]. However, it was found that the immunosuppressive effects of some highly chlorinated congeners might not involve AHR in mice (*Mus musculus*) [54]. In addition, the induction of EROD activity by 29 PCDEs was tested using a H4IIE rat hepatoma cell bioassay [108]. It was found that all the 29 PCDEs were inactive except for CDE 156 as a weak EROD inducer with a TEF value of approximately 1.2 × 10^−5^. The controversial results may be attributed to differences in experimental method and species sensitivity. Overall, the mechanisms of toxicities of PCDEs, especially whether the toxicities are mediated by AHR, need further research.

## 6. Future Perspectives

Future research may be carried out from the following aspects. First, more PCDE standards without PCDD/Fs impurities should be synthetized as a research basis. Second, the research on the environmental behavior of PCDEs on living organisms in real ecosystems, as well as exposure levels of PCDEs in wild animals and humans is still limited and needs to be strengthened. Third, chronic and multigenerational toxicological and ecotoxicological studies at environmentally relevant concentrations are needed to assess the impacts of long-term low-concentration exposure on individuals and populations. Fourth, previous studies mainly focused on the determination of basic acute toxicity endpoints. Moreover, there are conflicting views on whether the toxic effects are mediated by AHR. Thus, the molecular toxic mechanisms of PCDEs deserve more research by use of computational techniques, such as molecular docking and molecular dynamics simulations [113,114], as well as experimental methods. Fifth, inter-species sensitivity variations to PCDEs and effects of exposure at different developmental stages of organisms on population are also interesting. Finally, toxicity prediction models for identifying PCDE congeners of high priority and assessing health and ecological risks should be built in the future as soon as possible.

## 7. Conclusions

The existing studies on PCDEs from the perspectives of source, physicochemical property, environmental level and fate, synthesis and analysis and toxicology are reviewed in the present paper. These studies indicate that PCDEs have widely existed in various environmental media and organisms and can bioaccumulate and bioamplify through the food chain due to the long-range transport potential and high lipophilicity. PCDEs can be metabolized into other organic pollutants, such as HO-PCDEs, methoxylated PCDEs (MeO-PCDEs) and even PCDD/Fs through biotransformation, photolysis and pyrolysis reactions in the environment. In addition, the commonly used synthesis and analysis methods are coupling reaction and GC-MS/HRGC-HRMS, respectively. Toxicological studies have shown that PCDEs can cause lethality, teratogenicity, growth inhibition, tissue damage, reproductive and developmental abnormalities, oxidative stress, immunotoxicity and endocrine disorders in organisms. However, due to the lack of exposure and toxicological data, the health and ecological risk assessment of PCDEs has not yet been evaluated. Therefore, future research perspectives are also proposed to facilitate the assessment of health and ecological risks of PCDEs.

## Figures and Tables

**Table 1:** PCDE levels in water, sediment, soil and atmosphere.

Environmental Samples	Site	Ranges/Sums of PCDEs												Ref.
		DE	Mono-	Di-	Tri-	Tetra-	Penta-	Hexa-	Hepta-	Qcta-	Nona-	Deca-	∑PCDE	
**Water**														
At two meters of water on Pringle Cr. Bridge	Whitby Harbour (ng/L), Canada												1.57	[11]
At two meters of water on Whitby Harbour													0.68–7.07	
Surface water	Chaohu Lake (ng/L), China	0.006–0.012	0.012–0.133	0.006–0.151	0.031–0.564	0.023–0.211	0.01–0.152					0.016–0.088	0.351–2.021	[12]
Low water periods	Yangtze River (ng/L), China												1150–1800	[13]
High water periods													730–1300	
**Sediment**														
Sediments collected in 1991	Gulf of Finland and near Gotland (ng/g dw)					<0.1 (ranges of Tetra to Hepta-CDEs)				<0.2 (ranges of Qcta to Nona-CDEs)		<0.4		[14]
Sediments collected in 1993	River kymijoki (ng/g dw), Finland					<1–2	<1–17	<1–110	<1–110	<1–110	<2	<2	130–554	[15]
Sediments collected in 1997	River kymijoki (ng/g dw), Finland						0.11–57.6	0.39–91.1	0.37–75.7	0.22–49.8			8.79–606	[16]
Sediments collected in 2001							4.868–5.847	4.390–43.134	7.750–9.126	2.242–9.210			85	[17]
Contaminated area	Whitby Harbour (ng/g dw), Canada												622–1929	[11]
Michigan	Great Lake (ng/g dw), US												0.01–0.07	[18]
Huron													0.00–0.67	
Erie													0.12–0.46	
Lake Ontario													0.87–5.54	
Low water periods	Yangtze River (g/kg dw), China												1.58–3.98	[13]
High water periods													1.24–3.48	
Surface sediments	Chaohu lake (ng/g dw), China		0.015–0.081	0.015–0.104	0.022–0.44	0.023–0.580	0.034–0.269					0.023–0.341	0.279–2.474	[12]
SPM			0.01–0.066	0.01–0.103	0.012–0.309	0.013–0.305	0.018–0.204					0.065–0.296	0.331–2.013	
SP of upper bay	Narragansett Bay (ppt dw)				0.03	0.06							0.09	[19]
**Soil**														
Five contaminated sawmill sites	Sweden (ng/g dw)												<38–6800	[20]
**Atmosphere**														
Oceanic atmosphere	Taiwan and the northern Philippines (pg/m^3^)												0.00875	[21]
Rural area atmosphere													0.0144	
Stack flue gases	Electric arc furnaces (ng/nm^3^), China												0.0115	[22]

dw: dry weight; ppt: part per trillion.

**Table 2:** Concentrations of PCDEs detected in organisms.

Biological Organism	Site	Ranges/Sums of PCDEs											Ref.
		Mono-	Di-	Tri-	Tetra-	Penta-	Hexa-	Hepta-	Qcta-	Nona-	Deca-	∑PCDE	
**Invertebrates**													
Plankton	Whitby Harbour (ng/g ww)											6600–33,000	[11]
Mussel	Kymijoki River (ng/g lw)					0.42–1.2	0.78–1.1	0.36–0.89	0.4–0.71			4.71–5.67	[23]
	Narragansett Bay (ng/g dw)			3.6–125	24–416							27.6–541	[19]
Clam	Narragansett Bay (ng/g dw)			2.4	9.1							11.5	[19]
Oligochaetes worms	Downstream of the Ky-5 production plant in Kymijoki River (ng/g lw)					2.9–244	14.4–339	7.7–162	12.1–114			215–1325	[16]
	Whitby Harbour (ng/g ww)											5123–34,947	[11]
Chironomid larvae	Downstream of the Ky-5 production plant in Kymijoki River (ng/g lw)					2.7–296	1.1–265	1.63–76	0.79–72.5			0–1200	[16]
	Whitby Harbour (ng/g ww)											4042–29,292	[11]
Lobster	Narragansett Bay (ng/g dw)			82	144							226	[19]
Gammarus	Whitby Harbour (ng/g ww)											32,333–66,667	[11]
**Birds**													
Common tern	Rhode Island, US (ng/g ww)			130–280	450–900								[24]
Common tern egg				19–240	60–750								
Black skimmer egg	Louisiana, US (ng/g ww)				26–350								[24]
Bald eagle egg	Michigan, US (ng/g ww)					11–54							[24]
	Ohio, US (ng/g ww)					15							
White–tailed sea eagles	Gulf of Bothnia (ng/g lw)				<5–1300	7.8–1400	<10–7800	13–2200	13–13,000	27–1300	<20–270	1027–50,924	[25]
Black guillemots					<3–75	<3–53	<3–79	2.9–13	<3–9.3	<5–<10	<5–<10	233–354	
Black–crowned night heron eggs	Tianmu Lake (ng/g lw)						5.3	5.1	ND-37	11–340	ND-50	11–450	[26]
Whiskered tern eggs	East Tai Lake (ng/g lw)						ND-40	ND-50	ND-92	14–420	ND-73	15–700	[26]
**Fish**													
Pike	Kymijoki River (ng/g lw)				1–4	2–47	0–140	2–13	3–7			278–308	[27]
					<5–7	<7–48	<10–160	<10–93	6–88	<24–<25	<24–<25	677–706	[15]
	Whitby Harbour (ng/g)				10–461	3–524	7–874	9–409			2	1053–1803	[28]
Lake bream	Kymijoki River (ng/g lw)				5–6	2–72	12–250	18	23			642	[27]
Lake trout	Lake Ontario (ng/g lw)				TR-12	1.6–21.4	TR-34.2	0.8–35.9	0.6–10.9	0.6–2.6	0.9–5.8	54.2–105.6	[29]
	Lake Huron (ng/g lw)				ND-0.4	TR-2.2	TR-5.6	TR-4.7	0.6–1.9	0.4–0.9		6.1–29.3	
	Lake Superior (ng/g lw)					TR-0.5	TR-1	TR-1.2	TR-1	TR-1.2	TR	1.7–6.9	
Walleye	Lake Erie (ng/g lw)				ND-0.4	TR-5.1	0.4–23.7	0.6–17.4	0.4–2.9	TR-0.8	TR	34.9–105.6	[29]
Salmon	Simojiki River (ng/g fw)				<0.02–2.4	0.02–0.87	0.01–0.35	0.02–0.07	0.03–<0.2			1.49–4.4	[30]
	Tenojoki River (ng/g fw)				<0.01–0.06	<0.01–0.08	<0.02–0.06	<0.03	<0.05			0.05–0.51	
	Lake Saimaa (ng/g fw)				<0.01–0.11	<0.01–0.22	0.02–0.28	<0.02–<0.03	0.03–0.08			0.33–1.1	
	Lower Fraser River (μg/kg w.w)											0.53	[31]
	Duwamish River (μg/kg ww)											0.24	
Common carp	Whitby Harbour (ng/g ww)				5–1505	3–1876	8–2273	5–1346			2–4	768–14,005	[28]
Common shiner	Whitby Harbour (ng/g ww)											100–2857	[11]
Rosyface shiner												23,231–43,231	
Spottail shiner												20,706–96,529	
Pumpkinseed												30,417–68,250	
Yellow perch												4200–130,333	
Brown bullhead												7538–213,231	
White sucker												116,714–174,571	
Northern pike												21,000–447,000	
Arctic cod	Vestertana Fjord (ng/g lw)				0.5–5.2	<0.4–2.8	<0.4–0.9	<0.4	<0.4	<1	<1	14.4	[32]
**Mammals and human**													
Seal	Lake Saimaa (ng/g lw)				<1–11	<1–18	<1–44	<1–140	<1–110	2–12	<2	217–459	[15]
	Lake Baikal (ng/g lw)				<1–7	<1–6	<1–13	<1–3	<1–1	<2	<2	60	
	Hornby Island (ng/g lw)					1	0.9–3.9	0.5–1.5				11.6	[33]
	Vancouver (ng/g lw)				0.5	0.7–0.8	0.9–2.6	1.4				8.9	
	Smith Island (ng/g lw)					0.7	0.5–2.3	0.3–0.7				6.5	
	Gertrude Island (ng/g lw)					2.2	1.7–8.6	1.3–1.8				21.07	
	Gulf of Finland (ng/g lw)				4.5–17	<0.4–21	<0.3–62	0.3–36	<0.3–59	<0.4–15		39.9–373.9	[14]
Finnish human	Finland (ng/g lw)				<0.5–<1	<0.5–7.9	<0.5–2.4	<0.5–2	<0.5–<1	2.4–6	<1–<3	4.1–18.2	[32]
Human adipose tissue	Cornwal (ng/g lw)									0.81–1.39	0.12–0.32	0.95–1.71	[34]
	London (ng/g lw)									0.55–1.48	0.12–0.39	0.67–1.87	
	St.Catharines (ng/g lw)									1.17–2.94	0.42–1.02	1.64–3.96	
	Toronto (ng/g lw)									0.76–2.93	0.09–0.77	0.85–3.49	
	Windsor (ng/g lw)									0.68–1.43	0.25–0.37	0.92–1.8	
	East–north central of US (pg/g lw)						1–10	2–10	10–200	200–1000	5–20	224–1215	[35]
	East–south central of US (pg/g lw)						1–6	2–5	10–100	80–1000	ND-10	93–1121	
	Middle Atlantic of US (pg/g lw)						2–5	3–7	10–200	200–1000	8–10	220–1220	
	Mountain of US (pg/g lw)						1–4	1–4	5–200	50–800	ND-6	61–1008	
	New England of US (pg/g lw)						2–20	10–70	40–100	30–600	ND-20	30–790	
	Pacific of US (pg/g lw)						1–10	2–5	30–200	30–1000		31–1215	
	South Atlantic of US (pg/g lw)						2–10	3–10	10–200	100–2000	7–9	112–2119	
	West–north central of US (pg/g lw)						1–10	2–10	3–200	100–2000		104–2220	
	West–south central of US (pg/g lw)						3–4	3–5	20–100	200–900		226–1006	
**Food or health Products**													
Cod liver oil	US (ng/g lw)		8–10	17–46	3–11	2–9	3–19	10–253	6–25	<1	<1	659	[9]
	Germany (ng/g lw)		2–4	2–19			2–4	1–2	3–6			49	
Tuna in vegetable oil	Catalonia, Spain (ng/kg fw)				0.2	0.2	1.5	0.4	1.0			3.3	[36]
Sardine in vegetable oil					5.0	5.2	27.2	16.7	17.9			71.9	
Mussel	Collected from local markets of Catalonia in 2000 (ng/kg fw)				0.7–4.6	6.5–12.1	39.8–76.8	4.4–5.7	8.1–8.4			59.8–107	[36]
Hake					0.3–1.5	2.3–12.8	18.4–270	10.6–150	14.4–272			45.9–707	
Sardine					4.8–5.9	9.8–115	209–1531	72.0–14	104–914			400–2707	
Meat	Collected from Catalonia in 2000 (ng/kg ww)											1.27–3.11	[37]
Fish and seafood												3.29–1553.8	
Vegetables												0.24–0.55	
Fruits												0.71–0.85	
Eggs												1.29–1.29	
Milk												0.54–0.63	
Dairy products												0.66–3.69	
Cereals												3.86–5.50	
Tubers												1.02–1.02	
Pulses												1.16–1.31	
Oil and fats												11–11	
Sardine	Collected from fish markets of Catalonia in 2005 (ng/kg ww)				57–221	190–485	455–822	366–604	193–285			1312–2417	[38]
Tuna					1.8–51	5.8–292	4.8–1170	3.4–1120	2.3–785			18–3418	
Anchovy					30–277	92–659	278–876	211–595	132–311			743–2718	
Mackerel					55–74	203–260	232–405	179–337	73–184			742–1238	
Swordfish					1.7–7.4	12–41	16–136	7.9–97	4.8–47			48–325	
Salmon					34–92	88–169	114–151	18–52	24–26			302–453	
Hake					4–20	22–99	74–351	60–312	33–134			208–916	
Red mullet					88–414	594–2390	1110–3890	962–3010	627–1780			3382–11,484	
Sole					3.1–31	3.9–55	3.7–103	2.9–91	3.1–52			17–323	
Cuttlefish					0.2–1.9	0.2–17	1.7–30	0.2–22	0.1–44			2.4–107	
Squid					4.9–160	33–385	124–628	130–486	60–244			409–1903	
Mussel					54–197	11–20	12–19	4.6–9.5	2.8–5.1			95–236	
Clam					2–34	1.5–20	2.5–10	1.1–4.3	1.1–4.1			8.2–67	
Shrimp					0.2–1.2	0.4–6.6	0.8–23.7	0.2–18	0.1–21			1.8–70	
Meat and meat products	Collected from Catalonia in 2006 (ng/kg fw)				0.1–0.8	0.1–0.4	0.1–0.4	0.1–1.3	0.2–1.6			0.6–3.2	[39]
Fish and seafood					0.5–258.6	2.5–1296.7	7.5–2316.7	3.0–1867.3	2.6–1349.0			27.6–7088.2	
Vegetables					0.1–1.2	0.1–0.1	0.1–0.1	0.1–0.1	0.1–0.2			0.3–1.7	
Tubers					0.1	0.1	0.1	0.1	0.3			0.8	
Fruits					0.1–0.1	0.1–0.1	0.1–0.1	0.1–0.1	0.1–0.2			0.3–0.5	
Eggs					0.3	0.3	0.3	0.3	0.5			1.5	
Milk					0.1–0.7	0	0–0.2	0	0.1–0.1			0.3–1.1	
Dairy products					0–0.4	0–0.4	0–1.3	0–0.4	0.1–0.9			0.3–3.5	
Cereals					0.3–2.6	0.1–0.2	0.1–0.2	0.1–0.2	0.2–0.3			0.9–3.2	
Pulses					0.1–0.3	0.1–0.1	0.1–0.1	0.1–0.1	0.2–0.2			0.7–0.8	
Oils and fats					1.3–2.6	1.3–1.3	1.3–2.9	1.3–1.3	2.5–2.6			7.5–9.2	
Bakery products					0.3–0.4	0.3–0.4	0.3–0.4	0.3–0.4	0.6–0.8			1.8–2.3	
Veal steak	Collected from Catalonia in 2007 (Sum concentrations ng/kg fw)				1.10	1.03	0.95	0.53	0.78			4.38	[40]
Loin of pork					0.8	1.1	0.58	0.18	0.33			2.98	
Chicken					0.9	1.3	1.03	0.18	0.18			3.58	
Lamb					0.95	0.68	0.68	0.25	0.25			2.80	
String bean					1.4	1.83	2.85	0.25	0.38			6.7	
Potato					1.58	0.25	0.25	0.25	0.25			2.58	
Rice					3.6	0.25	0.38	0.63	0.63			5.48	
Olive oil					35.2	55	65	2.2	1.8			100.7	

ND: not detected; TR: trace level; dw: dry weight; ww: wet weight; lw: lipid weight; fw: fresh weight.

**Table 3:** Content of PCDEs as impurities in commercial chlorophenols and their downstream products.

Product	Manufacturer/Origin	Detection Concentration of PCDEs (mg/kg ww)	Ref.
**Chemical raw materials**			
2,4,5-trichlorophenol	Fluka AG/Germany	4.4	[9]
2,3,4,6-tetrachlorophenol	Fluka AG/Germany	213	
2,4,6-trichlorophenol Potassium 2,4,6-Trichlorophenate Potassium 2,3,4,6-Tetrachlorophenate	Sweden	100–1000	[8]
**Wood preservatives**			
Dowicide G ^a^	Dow Sweden Ltd./Sweden	590	[20]
Ky-5 ^b^	Kymi/Kymmene Oy/Finland	15/80	[15,63]
K1 ^c^	Kymi/Kymmene Oy/Finland	90	[63]
K12 ^b^	Kymi/Kymmene Oy/Finland	853	
Sadolins PX 65 ^d^	Desowag-Bayer Holzschuz GmbH/Germany	33	[9]
Xyladecor ^d^	Sadolin GmbH/Germany	21	

^a^ The main ingredient is sodium pentachlorophenate; ^b^ The main component is 2,3,4,6-tetrachlorophenol; ^c^ The main ingredient is sodium 2,3,4,6-tetrachlorophenate; ^d^ Contains 10% pentachlorophenol; ww: wet weight.

## Data Availability

Not applicable.

## Supplementary Materials

The following supporting information can be downloaded at: https://www.mdpi.com/article/10.3390/ijerph20053982/s1. Table S1: The names, IUPAC numbers and CAS numbers of 209 PCDE congeners; Table S2: The experimental physicochemical properties of 106 PCDE congeners; Table S3: Summary of physicochemical properties of PCDEs predicted by QSPR models; Table S4: Summary of toxic effects of PCDEs in organisms; Table S5: LC_50_ or EC_50_ values of PCDE congeners based on acute toxicity tests.

## References

[1] Koistinen J. (2000). Polychlorinated Diphenyl Ethers (PCDE).

[2] Nevalainen T.J., Rissanen K. (1994). AM1 and single-crystal X-ray diffraction study of the conformational properties of chlorinated diphenyl ethers. J. Chem. Soc. Perkin Trans. 2.

[3] Becker M., Phillips T., Safe S. (1991). Polychlorinated diphenyl ethers—A review. Toxicol. Environ. Chem..

[4] Sundström G., Hutzinger O. (1976). The synthesis of chlorinated diphenyl ethers. Chemosphere.

[5] Albro P.W., Parker C.E. (1980). General approach to the fractionation and class determination of complex mixtures of chlorinated aromatic compounds. J. Chromatogr. A.

[6] Ningbo Inno Pharmchem Co., Ltd. Intermediates of Difenoconazole CAS: 119446-68-3. https://www.tfindia.com/34-dichlorodiphenyl-ether/.

[7] Domingo J.L. (2006). Polychlorinated diphenyl ethers (PCDEs): Environmental levels, toxicity and human exposure: A review of the published literature. Environ. Int..

[8] Nilsson C.A., Renberg L. (1974). Further studies on impurities in chlorophenols. J. Chromatogr. A.

[9] Kurz J., Ballschmiter K. (1995). Isomer-specific determination of 79 polychlorinated diphenyl ethers (PCDE) in cod liver oils, chlorophenols and in a fly ash. Fresenius’ J. Anal. Chem..

[10] Yang J.S., Lin S.L., Lin T.C., Wu Y.L., Wang L.C., Chang-Chien G.P. (2015). Emissions of polychlorinated diphenyl ethers from a municipal solid waste incinerator during the start-up operation. J. Hazard. Mater..

[11] Villeneuve J.Y., Niimi A.J., Metcalfe C.D. (1999). Distribution and Bioaccumulation of Chlorinated Diphenyl Ethers in a Contaminated Embayment of Lake Ontario. J. Great Lakes Res..

[12] Zhang X., Wang T., Gao L., Feng M., Qin L., Shi J., Cheng D. (2018). Polychlorinated diphenyl ethers (PCDEs) in surface sediments, suspended particulate matter (SPM) and surface water of Chaohu Lake, China. Environ. Pollut..

[13] Qin L., Feng M., Zhang X., Wang L., Wang Z. (2015). Occurrence of polychlorinated diphenyl ethers in Nanjing section of the Yangtze River: Level and distribution pattern. Environ. Sci. Pollut. Res..

[14] Koistinen J., Stenman O., Haahti H., Suonperä M., Paasivirta J. (1997). Polychlorinated diphenyl ethers, dibenzo-p-dioxins, dibenzofurans and biphenyls in seals and sediment from the gulf of finland. Chemosphere.

[15] Koistinen J., Paasivirta J., Suonpera M., Hyvarinen H. (1995). Contamination of Pike and Sediment from the Kymijoki River by PCDEs, PCDDs, and PCDFs: Contents and Patterns Compared to Pike and Sediment from the Bothnian Bay and Seals from Lake Saimaa. Environ. Sci. Technol..

[16] Lyytikäinen M., Rantalainen A.L., Mikkelson P., Hämäläinen H., Paasivirta J., Kukkonen J. (2003). Similarities in bioaccumulation patterns of polychlorinated dibenzo-p-dioxins and furans and polychlorinated diphenyl ethers in laboratory-exposed oligochaetes and semipermeable membrane devices and in field-collected chironomids. Toxicol Environ. Chem. SETAC.

[17] Sormunen A.J., Koistinen J., Leppänen M.T., Kukkonen J.V.K. (2008). Desorption of sediment-associated polychlorinated dibenzo-p-dioxins, dibenzofurans, diphenyl ethers and hydroxydiphenyl ethers from contaminated sediment. Chemosphere.

[18] Li A., Guo J., Li Z., Lin T., Zhou S., He H., Ranansinghe P., Sturchio N.C., Rockne K.J., Giesy J.P. (2018). Legacy polychlorinated organic pollutants in the sediment of the Great Lakes. J. Great Lakes Res..

[19] Lake J.L., Rogerson P.F., Norwood C.B. (1981). A polychlorinated dibenzofuran and related compounds in an estuarine ecosystem. Environ. Sci. Technol..

[20] Persson Y., Lundstedt S., Öberg L., Tysklind M. (2007). Levels of chlorinated compounds (CPs, PCPPs, PCDEs, PCDFs and PCDDs) in soils at contaminated sawmill sites in Sweden. Chemosphere.

[21] Chao H.R., Lin D.Y., Chen K.Y., Gou Y.Y., Chiou T.H., Lee W.J., Chen S.J., Wang L.C. (2014). Atmospheric concentrations of persistent organic pollutants over the Pacific Ocean near southern Taiwan and the northern Philippines. Sci. Total Environ..

[22] Wu E.M.Y., Wang L.C., Lin S.L., Chang C.G.P. (2014). Validation and characterization of persistent organic pollutant emissions from stack flue gases of an electric arc furnace by using a long-term sampling system (AMESA^®^). Aerosol. Air Qual. Res..

[23] Koistinen J., Herve S., Paukku R., Lahtiperä M., Paasivirta J. (1997). Chloroaromatic pollutants in mussels incubated in two finnish watercourses polluted by industry. Chemosphere.

[24] Stafford C.J. (1983). Halogenated diphenyl ethers identified in avian tissues and eggs by GC/MS. Chemosphere.

[25] Koistinen J., Koivusaari J., Nuuja I., Paasivirta J. (1995). PCDEs, PCBs, PCDDs AND PCDFs in black guillemots and white-tailed sea eagles from the Baltic Sea. Chemosphere.

[26] Zhou Y., Yin G., Asplund L., Stewart K., Rantakokko P., Bignert A., Ruokojärvi P., Kiviranta H., Qiu Y., Ma Z. (2017). Human exposure to PCDDs and their precursors from heron and tern eggs in the Yangtze River Delta indicate PCP origin. Environ. Pollut..

[27] Koistinen J., Paasivirta J., Lahtiperä M. (1993). Bioaccumulation of dioxins, coplanar PCBs, PCDEs, HxCNs, R-PCNs, R-PCPHs and R-PCBBs in fish from a pulp-mill recipient watercourse. Chemosphere.

[28] Huestis S.Y., Sergeant D.B. (1992). Removal of chlorinated diphenyl ether interferences for analyses of PCDDs and PCDFs in fish. Chemosphere.

[29] Niimi A.J., Huestis S.Y., Metcalfe C.D. (1994). Chlorinated diphenyl ethers in Great Lakes fish and their environmental implication. Environ. Toxicol. Chem..

[30] Koistinen J., Vuorinen P.J., Paasivirta J. (1993). Contents and origin of polychlorinated diphenyl ethers (PCDE) in salmon from the Baltic Sea, Lake Saimaa and the Tenojoki river in Finland. Chemosphere.

[31] Cullon D.L., Yunker M.B., Alleyne C., Dangerfield N.J., O’Neill S., Whiticar M.J., Ross P.S. (2009). Persistent organic pollutants in chinook salmon (Oncorhynchus tshawytscha): Implications for resident killer whales of British Columbia and adjacent waters. Environ. Toxicol. Chem..

[32] Koistinen J., Mussalo-Rauhamaa H., Paasivirta J. (1995). Polychlorinated diphenyl ethers, dibenzo-p-dioxins and dibenzofurans in finnish human tissues compared to environmental samples. Chemosphere.

[33] Ross P.S., Noël M., Lambourn D., Dangerfield N., Calambokidis J., Jeffries S. (2013). Declining concentrations of persistent PCBs, PBDEs, PCDEs, and PCNs in harbor seals (*Phoca vitulina*) from the Salish Sea. Prog. Oceanogr..

[34] Williams D.T., Kennedy B., LeBel G.L. (1991). Chlorinated diphenyl ethers in human adipose tissue. Part 2. Chemosphere.

[35] Stanley J.S., Cramer P.H., Thornburg K.R., Remmers J.C., Breen J.J., Schwemberger J. (1991). Mass spectral confirmation of chlorinated and brominated diphenylethers in human adipose tissues. Chemosphere.

[36] Bocio A., Llobet J.M., Domingo J.L. (2004). Human Exposure to Polychlorinated Diphenyl Ethers through the Diet in Catalonia, Spain. J. Agric. Food Chem..

[37] Falcó G., Bocio A., Llobet J.M., Domingo J.L. (2005). Health risks of dietary intake of environmental pollutants by elite sportsmen and sportswomen. Food Chem. Toxicol..

[38] Domingo J.L., Bocio A., Falcó G., Llobett J.M. (2006). Exposure to PBDEs and PCDEs associated with the consumption of edible marine species. Environ. Sci. Technol..

[39] Martí-Cid R., Llobet J.M., Castell V., Domingo J.L. (2008). Human Exposure to Polychlorinated Naphthalenes and Polychlorinated Diphenyl Ethers from Foods in Catalonia, Spain: Temporal Trend. Environ. Sci. Technol..

[40] Perelló G., Martí-Cid R., Castell V., Llobet J.M., Domingo J.L. (2010). Influence of various cooking processes on the concentrations of PCDD/PCDFs, PCBs and PCDEs in foods. Food Control.

[41] Kurz J., Ballschmiter K. (1999). Vapour pressures, aqueous solubilities, Henry’s law constants, partition coefficients between gas/water (Kgw), N-octanol/water (Kow) and gas/N-octanol (Kgo) of 106 polychlorinated diphenyl ethers (PCDE). Chemosphere.

[42] Sinkkonen S., Paasivirta J. (2000). Polychlorinated organic compounds in the Arctic cod liver: Trends and profiles. Chemosphere.

[43] Niimi A.J. (1986). Biological half-lives of chlorinated diphenyl ethers in rainbow trout (*Salmo gairdneri*). Aquat. Toxicol..

[44] Opperhuizen A., Voors P.I. (1987). Bioconcentration kinetics of 2,4,5- tri- and 3,3′,4,4′-tetrachlorobiphenyl and 2,4,5- tri- and 3,3′,4,4′-tetrachlorodiphenylether in fish. Chemosphere.

[45] Chui Y.C., Addison R.F., Law F.C. (1990). Acute toxicity and toxicokinetics of chlorinated diphenyl ethers in trout. Xenobiotica.

[46] Zitko V., Carson W.G. (1977). Uptake and excretion of chlorinated diphenyl ethers and brominated toluenes by fish. Chemosphere.

[47] Neely W.B., Branson D.R., Blau G.E. (1974). Partition coefficient to measure bioconcentration potential of organic chemicals in fish. Environ. Sci. Technol..

[48] Lyytikäinen M., Hirva P., Minkkinen P., Hämäläinen H., Rantalainen A.L., Mikkelson P., Paasivirta J., Kukkonen J.V.K. (2003). Bioavailability of Sediment-Associated PCDD/Fs and PCDEs:  Relative Importance of Contaminant and Sediment Characteristics and Biological Factors. Environ. Sci. Technol..

[49] Qin L., Liu F., Liu H., Wei Z., Sun P., Wang Z. (2014). Evaluation of HODE-15, FDE-15, CDE-15, and BDE-15 toxicity on adult and embryonic zebrafish (*Danio rerio*). Environ. Sci. Pollut. Res..

[50] Metcalfe C.D., Metcalfe T.L., Cormier J.A., Huestis S.Y., Niimi A.J. (1997). Early life-stage mortalities of Japanese medaka (Oryzias latipes) exposed to polychlorinated diphenyl ethers. Environ. Toxicol. Chem..

[51] Zhang X., Feng M., Liu F., Qin L., Qu R., Li D., Wang Z. (2014). Subacute oral toxicity of BDE-15, CDE-15, and HODE-15 in ICR male mice: Assessing effects on hepatic oxidative stress and metals status and ascertaining the protective role of vitamin E. Environ. Sci. Pollut. Res. Int..

[52] Rosiak K., Li M.H., Degitz S.J., Skalla D.W., Chu I., Francis B.M. (1997). Maternal and developmental toxicity of polychlorinated diphenyl ethers (PCDEs) in Swiss-Webster mice and Sprague-Dawley rats. Toxicology.

[53] Rosiak K.L., Seo B.W., Chu I., Francis B.M. (1997). Effects of maternal exposure to chlorinated diphenyl ethers on thyroid hormone concentrations in maternal and juvenile rats. J. Environ. Sci. Health Part B.

[54] Harper N., Howie L., Connor K., Arellano L., Craig A., Dickerson R., Safe S. (1993). Immunosuppressive and Monooxygenase Induction Activities of Highly Chlorinated Diphenyl Ether Congeners in C57BL/6 and DBA/2 Mice. Fundam. Appl. Toxicol..

[55] Safe S. (1990). Polychlorinated biphenyls (PCBs), dibenzo-p-dioxins (PCDDs), dibenzofurans (PCDFs), and related compounds: Environmental and mechanistic considerations which support the development of toxic equivalency factors (TEFs). Crit. Rev. Toxicol..

[56] Howie L., Dickerson R., Davis D., Safe S. (1990). Immunosuppressive and monooxygenase induction activities of polychlorinated diphenyl ether congeners in C57BL6N mice: Quantitative structure-activity relationships. Toxicol. Appl. Pharmacol..

[57] Kim S., Thiessen P.A., Bolton E.E., Chen J., Fu G., Gindulyte A., Han L., He J., He S., Shoemaker B.A. (2016). PubChem substance and compound databases. Nucleic Acids Res..

[58] Zhang X.S., Xiong W.L., Wu Q.X., Nian K.N., Pan X.X., Doug C. (2023). Bioaccumulation, trophic transfers and biotransformation of polychlorinated diphenyl ethers in an indoor simulated aquatic food chain. Environ. Sci. Technol..

[59] Yang W., Huang X., Wu Q., Shi J., Zhang X., Ouyang L., Crump D., Zhang X., Zhang R. (2022). Acute toxicity of polychlorinated diphenyl ethers (PCDEs) in three model aquatic organisms (*Scenedesmus obliquus*, *Daphnia magna*, and *Danio rerio*) of different trophic levels. Sci. Total Environ..

[60] Igbinosa E.O., Odjadjare E.E., Chigor V.N., Igbinosa I.H., Emoghene A.O., Ekhaise F.O., Igiehon N.O., Idemudia O.G. (2013). Toxicological profile of chlorophenols and their derivatives in the environment: The public health perspective. Sci. World J..

[61] Steiert J.G., Crawford R.L. (1985). Microbial degradation of chlorinated phenols. Trends Biotechnol..

[62] World Health Organization, International Programme on Chemical Safety (1989). Chlorophenols Other Than Pentachlorophenol/Published Under the Joint Sponsorship of the United Nations Environment Programme, the International Labour Organisation, and the World Health Organization.

[63] Passivirta J., Lahtiperä M., Leskijärvi T. (1982). Experiences of structure analyses of chlorophenol dimers and trimers found in different samples. Chlorinated Dioxins Relat. Compd..

[64] Nakao T., Aozasa O., Ohta S., Miyata H. (2006). Formation of toxic chemicals including dioxin-related compounds by combustion from a small home waste incinerator. Chemosphere.

[65] Cheruiyot N.K., Yang H.H., Wang L.C., Lin C.C. (2020). Feasible and effective control strategies on extreme emissions of chlorinated persistent organic pollutants during the start-up processes of municipal solid waste incinerators. Environ. Pollut..

[66] Liu W., Zheng M., Liu W., Gao L., Su G., Zhang B. (2011). Mechanism of polychlorinated diphenyl ether formation on a simulated fly ash surface. J. Hazard. Mater..

[67] Liu W., Shen L., Zhang F., Liu W., Zheng M., Yang X. (2013). Influence of iron and copper oxides on polychlorinated diphenyl ether formation in heterogeneous reactions. Environ. Sci. Pollut. Res..

[68] Kurz J., Ballschmiter K. (1994). Relationship between structure and retention of polychlorinated diphenyl ethers (PCDE) in HRGC in comparison with other groups of halogenated aromatic compounds. Fresenius’ J. Anal. Chem..

[69] Yang P., Chen J., Chen S., Yuan X., Schramm K.W., Kettrup A. (2003). QSPR models for physicochemical properties of polychlorinated diphenyl ethers. Sci. Total Environ..

[70] Xu H.Y., Zou J.W., Hu G.X., Wang W. (2010). QSPR/QSAR models for prediction of the physico-chemical properties and biological activity of polychlorinated diphenyl ethers (PCDEs). Chemosphere.

[71] Sun L., Zhou L., Yu Y., Lan Y., Li Z. (2007). QSPR study of polychlorinated diphenyl ethers by molecular electronegativity distance vector (MEDV-4). Chemosphere.

[72] Zeng X., Wang Z., Ge Z., Liu H. (2007). Quantitative structure–property relationships for predicting subcooled liquid vapor pressure (PL) of 209 polychlorinated diphenyl ethers (PCDEs) by DFT and the position of Cl substitution (PCS) methods. Atmos. Environ..

[73] Yuan Y., Sun Y., Wang D., Liu R., Gu S., Liang G., Xu J. (2015). Quantitative structure-property relationship study of liquid vapor pressures for polychlorinated diphenyl ethers. Fluid Phase Equilib..

[74] Xiao F., Gulliver J.S., Simcik M.F. (2013). Predicting aqueous solubility of environmentally relevant compounds from molecular features: A simple but highly effective four-dimensional model based on Project to Latent Structures. Water Res..

[75] Cheng D., Cao K., Wang T., Zhang X., Feng M., Liu H. (2019). Evaluation of the oxidative stress in liver of crucian carp (*Carassius auratus*) exposed to 3,4,4′-tri-CDE, 2-MeO-3′,4,4′-tri-CDE, and 2-HO-3′,4,4′-tri-CDE. Environ. Sci. Pollut. Res. Int..

[76] Coburn J.A., Comba M. (1981). Identification of Polychlorinated Diphenyl Ethers in Whitby Harbour Sediments.

[77] He W., Qin N., Kong X., Liu W., He Q., Ouyang H., Wang Q., Yang B., Yang C., Jiang Y. (2013). Polybrominated diphenyl ethers (PBDEs) in the surface sediments and suspended particulate matter (SPM) from Lake Chaohu, a large shallow Chinese lake. Sci. Total Environ..

[78] Huo S., Li C., Xi B., Yu Z., Yeager K.M., Wu F. (2017). Historical record of polychlorinated biphenyls (PCBs) and special occurrence of PCB 209 in a shallow fresh-water lake from eastern China. Chemosphere.

[79] Martí-Cid R., Bocio A., Llobet J.M., Domingo J.L. (2007). Intake of chemical contaminants through fish and seafood consumption by children of Catalonia, Spain: Health risks. Food Chem. Toxicol..

[80] Domingo J.L. (2016). Nutrients and Chemical Pollutants in Fish and Shellfish. Balancing Health Benefits and Risks of Regular Fish Consumption. Crit. Rev. Food Sci. Nutr..

[81] Niimi A.J. (1996). Evaluation of PCBs and PCDDFs retention by aquatic organisms. Sci. Total Environ..

[82] Wu J.P., Luo X.J., Zhang Y., Yu M., Chen S.J., Mai B.X., Yang Z.Y. (2009). Biomagnification of polybrominated diphenyl ethers (PBDEs) and polychlorinated biphenyls in a highly contaminated freshwater food web from South China. Environ. Pollut..

[83] Komsta E., Chu I., Villeneuve D.C., Benoit F.M., Murdoch D. (1988). Tissue distribution metabolism and excretion of 2,2′,4,4′, 5-pentachlorodiphenyl ether in the rat. Arch. Toxicol..

[84] Tulp M.T.M., Sundström G., Martron L.B.J.M., Hutzinger O. (1979). Metabolism of Chlorodiphenyl Ethers and Irgasan^®^ DP 300. Xenobiotica.

[85] McClelland H.E., Jurs P. (2000). Quantitative Structure-Property Relationships for the Prediction of Vapor Pressures of Organic Compounds from Molecular Structures. J. Chem. Inf. Comput. Sci..

[86] Fischer R.C., Wittlinger R., Ballschmiter K. (1992). Retention-index based vapor pressure estimation for polychlorobiphenyl (PCB) by gas chromatography. Fresenius’ J. Anal. Chem..

[87] Akimoto Y., Nito S.i., Inouye Y. (1997). Comparative study on formations of polychlorinated dibenzo-p-dioxins, polychlorinated dibenzofurans and related compounds in a fluidized bed solid waste incinerator using long term used sand and fresh sand. Chemosphere.

[88] Wang S., Hao C., Gao Z., Chen J., Qiu J. (2014). Theoretical investigations on direct photolysis mechanisms of polychlorinated diphenyl ethers. Chemosphere.

[89] Altarawneh M., Dlugogorski B.Z. (2014). Mechanisms of transformation of polychlorinated diphenyl ethers into polychlorinated dibenzo-p-dioxins and dibenzofurans. Chemosphere.

[90] Choudhry G.G., Sundstroem G., Ruzo L.O., Hutzinger O. (1977). Photochemistry of chlorinated diphenyl ethers. J. Agric. Food Chem..

[91] Lindahl R., Rappe C., Buser H.R. (1980). Formation of polychlorinated dibenzofurans (PCDFs) and polychlorinated dibenzo-p-dioxins (PCDDs) from the pyrolysis of polychlorinated diphenyl ethers. Chemosphere.

[92] Norström Å., Andersson K., Rappe C. (1976). Formation of chlorodibenzofurans by irradiation of chlorinated diphenyl ethers. Chemosphere.

[93] Norström Å., Andersson K., Rappe C. (1976). Palladium (II) acetate promoted cyclization of polychlorinated diphenyl ethers to the corresponding dibenzofurans. Chemosphere.

[94] Garå A., Nilsson C.A., Andersson K., Rappe C. (1979). Synthesis of higher chlorinated dibenzofurans. Chemosphere.

[95] Nevalainen T., Koistinen J., Nurmela P. (1994). Synthesis, Structure Verification, and Chromatographic Relative Retention Times for Polychlorinated Diphenyl Ethers. Environ. Sci. Technol..

[96] Nilsson C.A., Norström Å. (1977). The synthesis of halogenated diphenyliodonium salts and their coupling products with halogenated phenols. Chemosphere..

[97] Zhang X., Liu F., Wei Z., Wang Z. (2013). Synthesis of Diaryl Ethers by CuI-Catalyzed C-O Bond Formation via Ullman Coupling: Assessing the Reactivity of Aryl Halides. Lett. Org. Chem..

[98] Čermák J.K., Církva V. (2014). Copper-mediated synthesis of mono- and dichlorinated diaryl ethers. Tetrahedron Lett..

[99] Chan D.M.T., Monaco K.L., Wang R.P., Winters M.P. (1998). New N- and O-arylations with phenylboronic acids and cupric acetate. Tetrahedron Lett..

[100] Newsome W.H., Shields J.B. (1982). Method for the determination of higher chlorinated diphenyl ethers in chicken tissue. J. Chromatogr..

[101] Kuehl D.W., Durhan E.J., Butterworth B.C., Linn D. (1984). Identification of polychlorinated planar chemicals in fishes from major watersheds near the Great Lakes. Environ. Int..

[102] Villanueva E.C., Burse V.W., Jennings R.W. (1973). Chlorodibenzo-p-dioxin contamination of two commercially available pentachlorophenols. J. Agric. Food Chem..

[103] Chang Y.C., Lee W.J., Yang H.H., Wang L.C., Lu J.H., Tsai Y.I., Cheng M.T., Young L.H., Chiang C.J. (2014). Reducing Emissions of Persistent Organic Pollutants from a Diesel Engine by Fueling with Water-Containing Butanol Diesel Blends. Environ. Sci. Technol..

[104] Fang B., Shi J., Qin L., Feng M., Cheng D., Wang T., Zhang X. (2018). Toxicity evaluation of 4,4′-di-CDPS and 4,4′-di-CDE on green algae *Scenedesmus obliquus*: Growth inhibition, change in pigment content, and oxidative stress. Environ. Sci. Pollut. Res. Int..

[105] Chu I., Villeneuve D.C., Secours V., Valli V.E. (1989). Toxicological assessment of chlorinated diphenyl ethers in the rat. J. Environ. Sci. Health Part B.

[106] Chu I., Villeneuve D.C., Secours V., Valli V.E. (1990). Toxicological assessment of chlorinated diphenyl ethers in the rat, Part II. J. Environ. Sci. Health Part B.

[107] Ye C., Xiong W., Shi S., Shi J., Yang W., Zhang X. (2022). Biomarker Responses, Gene Expression Alterations, and Histological Changes in Zebrafish (*Danio rerio*) After In Vivo Exposure to Polychlorinated Diphenyl Ethers. Front. Physiol..

[108] Koistinen J., Sanderson J.T., Giesy J.P., Nevalainen T., Paasivirta J. (1996). Ethoxyresorufin-O-deethylase induction potency of polychlorinated diphenyl ethers in H4IIE rat hepatoma cells. Environ. Toxicol. Chem..

[109] Pastershank G.M., Kiparissis Y., Metcalfe C.D. (1999). Induction of hepatic ethoxyresorufin-O-deethylase (EROD) in rainbow trout (Oncorhynchus mykiss) exposed to 3,3′,4,4′-tetrachlorodiphenyl ether by intraperitoneal injection or gavage intubation. Chemosphere.

[110] Iverson F., Newsome H., Hierlihy L. (1987). Induction of rat hepatic monooxygenase activity by polychlorinated diphenyl ethers. Food Chem. Toxicol..

[111] Nevalainen T., Kolehmainen E. (1994). New qsar models for polyhalogenated aromatics. Environ. Toxicol. Chem..

[112] Chui Y.C., Hansell M.M., Addison R.F., Law F.C.P. (1985). Effects of chlorinated diphenyl ethers on the mixed-function oxidases and ultrastructure of rat and trout liver. Toxicol. Appl. Pharmacol..

[113] Wang X., Zhang R., Song C., Crump D. (2020). Computational evaluation of interactions between organophosphate esters and nuclear hormone receptors. Environ. Res..

[114] Wang X., Zhang X., Xia P., Zhang J., Wang Y., Zhang R., Giesy J.P., Shi W., Yu H. (2017). A high-throughput, computational system to predict if environmental contaminants can bind to human nuclear receptors. Sci. Total. Environ..

