# Supplementary material for: Polychlorinated Diphenyl Ethers in the Environment: A Review and Future Perspectives
Source: Int J Environ Res Public Health. 2023 Feb 23;20(5):3982. doi: 10.3390/ijerph20053982 (PMC10002337; doi:10.3390/ijerph20053982)
Supplement: Supplementary file 1 [file ijerph-20-03982-s001.zip › ijerph-2229115-Supplementary.pdf]

SUPPORTING INFORMATION

Polychlorinated Diphenyl Ethers in the Environment: A Review and Future Perspectives

Qiuxuan Wu [1], Qiong Wu [1], Xiaoxiang Wang [2], Xuesheng Zhang [3,4,*] and Rui Zhang [1,*]

[1] School of Water Conservancy and Environment, University of Jinan, Jinan 250022, China
[2] Institute for Carbon-Neutral Technology, Shenzhen Polytechnic, Shenzhen 518055, China
[3] School of Resources and Environmental Engineering, Anhui University, Hefei 230601, China
[4] Laboratory of Wetland Protection and Ecological Restoration, Anhui University, Hefei 230601, China
* Correspondence: zhangxs8725@ahu.edu.cn (X.Z.); stu_zhangr@ujn.edu.cn (R.Z.); Tel.: +86-551-63861985 (X.Z.); +86-531-82769233 (R.Z.)

Number of pages: 15; Number of tables: 5.

Table S1. The names, IUPAC numbers and CAS numbers of 209 PCDE congeners.

Compound	IUPAC number	CAS number
**Diphenyl ether**	CDE-0	101-84-8
**Monochlorodiphenyl ether**		
2-Monochlorodiphenyl ether	CDE-1	2689-07-8
3-Monochlorodiphenyl ether	CDE-2	6452-49-9
4-Monochlorodiphenyl ether	CDE-3	7005-72-3
**Dichlorodiphenyl ether**		
2,2'-Dichlorodiphenyl ether	CDE-4	7024-98-8
2,3-Dichlorodiphenyl ether	CDE-5	28675-08-3
2,3'-Dichlorodiphenyl ether	CDE-6	7024-99-9
2,4-Dichlorodiphenyl ether	CDE-7	51892-26-3
2,4'-Dichlorodiphenyl ether	CDE-8	6903-65-7
2,5-Dichlorodiphenyl ether	CDE-9	24910-69-8
2,6-Dichlorodiphenyl ether	CDE-10	28419-69-4
3,3'-Dichlorodiphenyl ether	CDE-11	6903-62-4
3,4-Dichlorodiphenyl ether	CDE-12	55538-69-7
3,4'-Dichlorodiphenyl ether	CDE-13	6842-62-2
3,5-Dichlorodiphenyl ether	CDE-14	24910-68-7
4,4'-Dichlorodiphenyl ether	CDE-15	2444-89-5
**Trichlorodiphenyl ether**		
2,2',3-Trichlorodiphenyl ether	CDE-16	727738-38-7
2,2',4-Trichlorodiphenyl ether	CDE-17	68914-97-6
2,2',5-Trichlorodiphenyl ether	CDE-18	727738-42-3
2,2',6-Trichlorodiphenyl ether	CDE-19	727738-44-5
2,3,3'-Trichlorodiphenyl ether	CDE-20	727738-39-8
2,3,4-Trichlorodiphenyl ether	CDE-21	85918-32-7
2,3,4'-Trichlorodiphenyl ether	CDE-22	157683-71-1
2,3,5-Trichlorodiphenyl ether	CDE-23	162853-24-9
2,3,6-Trichlorodiphenyl ether	CDE-24	162853-25-0
2,3',4-Trichlorodiphenyl ether	CDE-25	155999-93-2
2,3',5-Trichlorodiphenyl ether	CDE-26	727738-43-4
2,3',6-Trichlorodiphenyl ether	CDE-27	727738-45-6
2,4,4'-Trichlorodiphenyl ether	CDE-28	59039-21-3

2,4,5-Trichlorodiphenyl ether	CDE-29	52322-80-2
2,4,6-Trichlorodiphenyl ether	CDE-30	63646-52-6
2,4',5-Trichlorodiphenyl ether	CDE-31	65075-00-5
2,4',6-Trichlorodiphenyl ether	CDE-32	157683-72-2
2',3,4-Trichlorodiphenyl ether	CDE-33	61328-44-7
2',3,5-Trichlorodiphenyl ether	CDE-34	727738-40-1
3,3',4-Trichlorodiphenyl ether	CDE-35	66794-60-3
3,3',5-Trichlorodiphenyl ether	CDE-36	727738-41-2
3,4,4'-Trichlorodiphenyl ether	CDE-37	63646-51-5
3,4,5-Trichlorodiphenyl ether	CDE-38	63646-53-7
3,4',5-Trichlorodiphenyl ether	CDE-39	24910-73-4
**Tetrachlorodiphenyl ether**		
2,2',3,3'-Tetrachlorodiphenyl ether	CDE-40	727738-46-7
2,2',3,4-Tetrachlorodiphenyl ether	CDE-41	220002-37-9
2,2',3,4'-Tetrachlorodiphenyl ether	CDE-42	147102-63-4
2,2',3,5-Tetrachlorodiphenyl ether	CDE-43	727738-50-3
2,2',3,5'-Tetrachlorodiphenyl ether	CDE-44	727738-47-8
2,2',3,6-Tetrachlorodiphenyl ether	CDE-45	727738-52-5
2,2',3,6'-Tetrachlorodiphenyl ether	CDE-46	727738-48-9
2,2',4,4'-Tetrachlorodiphenyl ether	CDE-47	28076-73-5
2,2',4,5-Tetrachlorodiphenyl ether	CDE-48	162853-26-1
2,2',4,5'-Tetrachlorodiphenyl ether	CDE-49	155999-92-1
2,2',4,6-Tetrachlorodiphenyl ether	CDE-50	727738-54-7
2,2',4,6'-Tetrachlorodiphenyl ether	CDE-51	147102-65-6
2,2',5,5'-Tetrachlorodiphenyl ether	CDE-52	727738-56-9
2,2',5,6'-Tetrachlorodiphenyl ether	CDE-53	727738-57-0
2,2',6,6'-Tetrachlorodiphenyl ether	CDE-54	91371-42-5
2,3,3',4-Tetrachlorodiphenyl ether	CDE-55	220002-39-1
2,3,3',4'-Tetrachlorodiphenyl ether	CDE-56	162853-27-2
2,3,3',5-Tetrachlorodiphenyl ether	CDE-57	727738-51-4
2,3,3',5'-Tetrachlorodiphenyl ether	CDE-58	727738-49-0
2,3,3',6-Tetrachlorodiphenyl ether	CDE-59	727738-53-6
2,3,4,4'-Tetrachlorodiphenyl ether	CDE-60	65075-01-6
2,3,4,5-Tetrachlorodiphenyl ether	CDE-61	220002-40-4
2,3,4,6-Tetrachlorodiphenyl ether	CDE-62	85918-33-8

2,3,4',5-Tetrachlorodiphenyl ether	CDE-63	220002-41-5
2,3,4',6-Tetrachlorodiphenyl ether	CDE-64	220002-42-6
2,3,5,6-Tetrachlorodiphenyl ether	CDE-65	63646-54-8
2,3',4,4'-Tetrachlorodiphenyl ether	CDE-66	61328-46-9
2,3',4,5-Tetrachlorodiphenyl ether	CDE-67	152833-52-8
2,3',4,5'-Tetrachlorodiphenyl ether	CDE-68	147102-64-5
2,3',4,6-Tetrachlorodiphenyl ether	CDE-69	727738-55-8
2,3'4',5-Tetrachlorodiphenyl ether	CDE-70	159553-67-0
2,3',4',6-Tetrachlorodiphenyl ether	CDE-71	130892-66-9
2,3',5,5'-Tetrachlorodiphenyl ether	CDE-72	727738-58-1
2,3',5',6-Tetrachlorodiphenyl ether	CDE-73	727738-59-2
2,4,4',5-Tetrachlorodiphenyl ether	CDE-74	61328-45-8
2,4,4',6-Tetrachlorodiphenyl ether	CDE-75	63553-30-0
2,3',4',5'-Tetrachlorodiphenyl ether	CDE-76	727738-60-5
3,3',4,4'-Tetrachlorodiphenyl ether	CDE-77	56348-72-2
3,3',4,5-Tetrachlorodiphenyl ether	CDE-78	727738-61-6
3,3',4,5'-Tetrachlorodiphenyl ether	CDE-79	552884-22-7
3,3',5,5'-Tetrachlorodiphenyl ether	CDE-80	85918-34-9
3,4,4',5-Tetrachlorodiphenyl ether	CDE-81	62615-07-0
**Pentachlorodiphenyl ether**		
2,2',3,3',4-Pentachlorodiphenyl ether	CDE-82	160282-10-0
2,2',3,3',5-Pentachlorodiphenyl ether	CDE-83	727738-65-0
2,2',3,3',6-Pentachlorodiphenyl ether	CDE-84	727738-71-8
2,2',3,4,4'-Pentachlorodiphenyl ether	CDE-85	71585-37-0
2,2',3,4,5-Pentachlorodiphenyl ether	CDE-86	727738-62-7
2,2',3,4,5'-Pentachlorodiphenyl ether	CDE-87	160282-09-7
2,2',3,4,6-Pentachlorodiphenyl ether	CDE-88	182346-19-6
2,2',3,4,6'-Pentachlorodiphenyl ether	CDE-89	85918-35-0
2,2',3,4',5-Pentachlorodiphenyl ether	CDE-90	157683-73-3
2,2',3,4',6-Pentachlorodiphenyl ether	CDE-91	116995-20-1
2,2',3,5,5'-Pentachlorodiphenyl ether	CDE-92	727738-66-1
2,2',3,5,6-Pentachlorodiphenyl ether	CDE-93	727738-69-4
2,2',3,5,6'-Pentachlorodiphenyl ether	CDE-94	727738-67-2
2,2',3,5',6-Pentachlorodiphenyl ether	CDE-95	727738-72-9
2,2',3,6,6'-Pentachlorodiphenyl ether	CDE-96	727738-73-0

2,2',3,4',5'-Pentachlorodiphenyl ether	CDE-97	160282-08-6
2,2',3,4',6'-Pentachlorodiphenyl ether	CDE-98	727738-75-2
2,2',4,4',5-Pentachlorodiphenyl ether	CDE-99	60123-64-0
2,2',4,4',6-Pentachlorodiphenyl ether	CDE-100	104294-16-8
2,2',4,5,5'-Pentachlorodiphenyl ether	CDE-101	131138-21-1
2,2',4,5,6'-Pentachlorodiphenyl ether	CDE-102	130892-67-0
2,2',4,5',6-Pentachlorodiphenyl ether	CDE-103	727738-76-3
2,2',4,6,6'-Pentachlorodiphenyl ether	CDE-104	727738-77-4
2,3,3',4,4'-Pentachlorodiphenyl ether	CDE-105	85918-31-6
2,3,3',4,5-Pentachlorodiphenyl ether	CDE-106	727738-63-8
2,3,3',4',5-Pentachlorodiphenyl ether	CDE-107	159553-68-1
2,3,3',4,5'-Pentachlorodiphenyl ether	CDE-108	160282-07-5
2,3,3',4,6-Pentachlorodiphenyl ether	CDE-109	727738-64-9
2,3,3',4',6-Pentachlorodiphenyl ether	CDE-110	159553-69-2
2,3,3',5,5'-Pentachlorodiphenyl ether	CDE-111	727738-68-3
2,3,3',5,6-Pentachlorodiphenyl ether	CDE-112	727738-70-7
2,3,3',5',6-Pentachlorodiphenyl ether	CDE-113	727738-74-1
2,3,4,4',5-Pentachlorodiphenyl ether	CDE-114	113464-17-8
2,3,4,4',6-Pentachlorodiphenyl ether	CDE-115	160282-05-3
2,3,4,5,6-Pentachlorodiphenyl ether	CDE-116	42279-29-8
2,3,4',5,6-Pentachlorodiphenyl ether	CDE-117	63646-55-9
2,3',4,4',5-Pentachlorodiphenyl ether	CDE-118	60123-65-1
2,3',4,4',6-Pentachlorodiphenyl ether	CDE-119	157683-74-4
2,3',4,5,5'-Pentachlorodiphenyl ether	CDE-120	160282-04-2
2,3',4,5',6-Pentachlorodiphenyl ether	CDE-121	76621-13-1
2,3,3',4',5'-Pentachlorodiphenyl ether	CDE-122	727738-78-5
2,3',4,4',5'-Pentachlorodiphenyl ether	CDE-123	160282-06-4
2,3',4',5,5'-Pentachlorodiphenyl ether	CDE-124	727738-79-6
2,3',4',5',6-Pentachlorodiphenyl ether	CDE-125	727738-80-9
3,3',4,4',5-Pentachlorodiphenyl ether	CDE-126	94339-59-0
3,3',4,5,5'-Pentachlorodiphenyl ether	CDE-127	220002-43-7
**Hexachlorodiphenyl ether**		
2,2',3,3',4,4'-Hexachlorodiphenyl ether	CDE-128	71585-39-2
2,2',3,3',4,5-Hexachlorodiphenyl ether	CDE-129	159553-70-5
2,2',3,3',4,5'-Hexachlorodiphenyl ether	CDE-130	76621-14-2

2,2',3,3',4,6-Hexachlorodiphenyl ether	CDE-131	727738-84-3
2,2',3,3',4,6'-Hexachlorodiphenyl ether	CDE-132	124076-66-0
2,2',3,3',5,5'-Hexachlorodiphenyl ether	CDE-133	727738-87-6
2,2',3,3',5,6-Hexachlorodiphenyl ether	CDE-134	727738-90-1
2,2',3,3',5,6'-Hexachlorodiphenyl ether	CDE-135	727738-88-7
2,2',3,3',6,6'-Hexachlorodiphenyl ether	CDE-136	117948-40-0
2,2',3,4,4',5-Hexachlorodiphenyl ether	CDE-137	71585-38-1
2,2',3,4,4',5'-Hexachlorodiphenyl ether	CDE-138	71585-36-9
2,2',3,4,4',6-Hexachlorodiphenyl ether	CDE-139	106220-83-1
2,2',3,4,4',6'-Hexachlorodiphenyl ether	CDE-140	106220-82-0
2,2',3,4,5,5'-Hexachlorodiphenyl ether	CDE-141	727738-81-0
2,2',3,4,5,6-Hexachlorodiphenyl ether	CDE-142	727738-82-1
2,2',3,4,5,6'-Hexachlorodiphenyl ether	CDE-143	159553-71-6
2,2',3,4,5',6-Hexachlorodiphenyl ether	CDE-144	727738-85-4
2,2',3,4,6,6'-Hexachlorodiphenyl ether	CDE-145	165282-28-0
2,2',3,4',5,5'-Hexachlorodiphenyl ether	CDE-146	162853-28-3
2,2',3,4',5,6-Hexachlorodiphenyl ether	CDE-147	116995-18-7
2,2',3,4',5,6'-Hexachlorodiphenyl ether	CDE-148	727738-89-8
2,2',3,4',5',6-Hexachlorodiphenyl ether	CDE-149	85918-37-2
2,2',3,4',6,6'-Hexachlorodiphenyl ether	CDE-150	116995-19-8
2,2',3,5,5',6-Hexachlorodiphenyl ether	CDE-151	727738-91-2
2,2',3,5,6,6'-Hexachlorodiphenyl ether	CDE-152	727738-92-3
2,2',4,4',5,5'-Hexachlorodiphenyl ether	CDE-153	71859-30-8
2,2',4,4',5,6'-Hexachlorodiphenyl ether	CDE-154	106220-81-9
2,2',4,4',6,6'-Hexachlorodiphenyl ether	CDE-155	6973-37-1
2,3,3',4,4',5-Hexachlorodiphenyl ether	CDE-156	109828-22-0
2,3,3',4,4',5'-Hexachlorodiphenyl ether	CDE-157	94339-60-3
2,3,3',4,4',6-Hexachlorodiphenyl ether	CDE-158	85918-36-1
2,3,3',4,5,5'-Hexachlorodiphenyl ether	CDE-159	159553-72-7
2,3,3',4,5,6-Hexachlorodiphenyl ether	CDE-160	727738-83-2
2,3,3',4,5',6-Hexachlorodiphenyl ether	CDE-161	727738-86-5
2,3,3',4',5,5'-Hexachlorodiphenyl ether	CDE-162	89026-26-6
2,3,3',4',5,6-Hexachlorodiphenyl ether	CDE-163	155999-97-6
2,3,3',4',5',6-Hexachlorodiphenyl ether	CDE-164	727738-93-4
2,3,3',5,5',6-Hexachlorodiphenyl ether	CDE-165	130480-89-6

2,3,4,4',5,6-Hexachlorodiphenyl ether	CDE-166	63646-56-0
2,3',4,4',5,5'-Hexachlorodiphenyl ether	CDE-167	131138-20-0
2,3',4,4',5',6-Hexachlorodiphenyl ether	CDE-168	727738-94-5
3,3',4,4',5,5'-Hexachlorodiphenyl ether	CDE-169	727738-95-6
**Heptachlorodiphenyl ether**		
2,2',3,3',4,4',5-Heptachlorodiphenyl ether	CDE-170	71585-40-5
2,2',3,3',4,4',6-Heptachlorodiphenyl ether	CDE-171	727738-99-0
2,2',3,3',4,5,5'-Heptachlorodiphenyl ether	CDE-172	83992-74-9
2,2',3,3',4,5,6-Heptachlorodiphenyl ether	CDE-173	727738-96-7
2,2',3,3',4,5,6'-Heptachlorodiphenyl ether	CDE-174	159553-73-8
2,2',3,3',4,5',6-Heptachlorodiphenyl ether	CDE-175	727739-00-6
2,2',3,3',4,6,6'-Heptachlorodiphenyl ether	CDE-176	727739-01-7
2,2',3,3',4',5,6-Heptachlorodiphenyl ether	CDE-177	83992-71-6
2,2',3,3',5,5',6-Heptachlorodiphenyl ether	CDE-178	727739-03-9
2,2',3,3',5,6,6'-Heptachlorodiphenyl ether	CDE-179	727739-04-0
2,2',3,4,4',5,5'-Heptachlorodiphenyl ether	CDE-180	83992-69-2
2,2',3,4,4',5,6-Heptachlorodiphenyl ether	CDE-181	157683-75-5
2,2',3,4,4',5,6'-Heptachlorodiphenyl ether	CDE-182	88467-63-4
2,2',3,4,4',5',6-Heptachlorodiphenyl ether	CDE-183	106220-85-3
2,2',3,4,4',6,6'-Heptachlorodiphenyl ether	CDE-184	106220-84-2
2,2',3,4,5,5',6-Heptachlorodiphenyl ether	CDE-185	727738-97-8
2,2',3,4,5,6,6'-Heptachlorodiphenyl ether	CDE-186	22274-48-2
2,2',3,4',5,5',6-Heptachlorodiphenyl ether	CDE-187	109828-23-1
2,2',3,4',5,6,6'-Heptachlorodiphenyl ether	CDE-188	116995-22-3
2,3,3',4,4',5,5'-Heptachlorodiphenyl ether	CDE-189	83992-72-7
2,3,3',4,4',5,6-Heptachlorodiphenyl ether	CDE-190	83992-70-5
2,3,3',4,4',5',6-Heptachlorodiphenyl ether	CDE-191	55684-92-9
2,3,3',4,5,5',6-Heptachlorodiphenyl ether	CDE-192	727738-98-9
2,3,3',4',5,5',6-Heptachlorodiphenyl ether	CDE-193	727739-05-1
**Octachlorodiphenyl ether**		
2,2',3,3',4,4',5,5'-Octachlorodiphenyl ether	CDE-194	57379-40-5
2,2',3,3',4,4',5,6-Octachlorodiphenyl ether	CDE-195	65075-02-7
2,2',3,3',4,4',5,6'-Octachlorodiphenyl ether	CDE-196	85918-38-3
2,2',3,3',4,4',6,6'-Octachlorodiphenyl ether	CDE-197	117948-62-6
2,2',3,3',4,5,5',6-Octachlorodiphenyl ether	CDE-198	152833-53-9

2,2',3,3',4,5,5',6'-Octachlorodiphenyl ether	CDE-199	83992-76-1
2,2',3,3',4,5,6,6'-Octachlorodiphenyl ether	CDE-200	727739-06-2
2,2',3,3',4,5',6,6'-Octachlorodiphenyl ether	CDE-201	116995-21-2
2,2,3,3',5,5',6,6'-Octachlorodiphenyl ether	CDE-202	727739-08-4
2,2',3,4,4',5,5',6-Octachlorodiphenyl ether	CDE-203	83992-75-0
2,2',3,4,4',5,6,6'-Octachlorodiphenyl ether	CDE-204	157683-76-6
2,3,3',4,4',5,5',6-Octachlorodiphenyl ether	CDE-205	727739-07-3
**Nonachlorodiphenyl ether**		
2,2',3,3',4,4',5,5',6-Nonachlorodiphenyl ether	CDE-206	83992-73-8
2,2',3,3',4,4',5,6,6'-Nonachlorodiphenyl ether	CDE-207	148934-69-4
2,2',3,3',4,5,5',6,6'-Nonachlorodiphenyl ether	CDE-208	148934-68-3
**Decachlorodiphenyl ether**		
2,2',3,3',4,4',5,5',6,6'-Decachlorodiphenyl ether	CDE-209	31710-30-2

Table S2. The experimental physicochemical properties of 106 PCDE congeners [41].

IUPAC number	-log P_L (Pa)	-log $S_{w,L}$ (mol/L)	H (Pa m^3/mol)	-log K_{gw} (25 °C)	log K_{ow}	-log K_{go}
CDE-0	-0.38	3.56	8.71	2.45	3.97	6.42
**Mono-CDE**						
CDE-1	0.27	4.78	32.36	1.88	4.45	6.33
CDE-2	0.30	4.21	8.13	2.48	4.75	6.93
CDE-3	0.36	4.32	9.12	2.43	4.70	7.13
**Di-CDE**						
CDE-5	1.05	4.67	4.17	2.77	5.00	7.77
CDE-7	0.91	4.63	5.25	2.67	4.93	7.60
CDE-8	1.03	5.52	30.90	1.90	5.03	6.93
CDE-9	0.87	4.97	12.59	2.29	5.13	7.42
CDE-10	0.76	5.06	19.95	2.09	4.64	6.73
CDE-12	1.06	4.72	9.12	2.43	4.99	7.42
CDE-13	1.06	4.77	7.94	2.49	5.13	7.62
CDE-14	0.87	5.06	15.49	2.20	5.21	7.42
CDE-15	1.13	4.80	4.68	2.72	5.25	7.97
**Tri-CDE**						
CDE-17	1.60	4.95	2.24	3.04	4.96	8.00
CDE-21	1.76	5.32	3.63	2.83	5.55	8.38
CDE-22	1.82	5.31	3.09	2.90	5.63	8.53
CDE-23	1.53	5.19	4.57	2.73	5.62	8.35
CDE-24	1.42	5.95	33.88	1.86	5.35	7.21
CDE-25	1.62	5.44	6.61	2.57	5.65	8.22
CDE-28	1.69	6.22	33.88	1.86	5.53	7.19
CDE-29	1.53	6.58	112.20	1.34	5.58	6.92
CDE-30	1.26	6.11	70.79	1.54	5.32	6.86
CDE-31	1.64	5.44	6.31	2.59	5.66	8.25
CDE-32	1.53	5.91	23.99	2.01	5.30	7.31
CDE-33	1.73	5.20	2.95	2.92	5.50	8.42
CDE-35	1.78	5.43	4.47	2.74	5.74	8.48
CDE-37	1.86	5.66	6.31	2.59	5.88	8.47
CDE-38	1.73	6.77	109.65	1.35	5.70	7.05
CDE-39	1.64	5.52	6.31	2.59	5.77	8.36

**Tetra-CDE**						
CDE-41	2.48	6.74	18.20	2.13	5.72	7.85
CDE-42	2.41	6.65	17.38	2.15	5.88	8.03
CDE-47	2.28	6.82	34.67	1.85	5.95	7.80
CDE-48	2.20	7.00	63.10	1.59	5.97	7.56
CDE-49	2.18	6.77	38.90	1.80	5.78	7.58
CDE-55	2.46	7.09	42.66	1.76	6.07	7.83
CDE-56	2.54	6.90	22.91	2.03	5.99	8.02
CDE-60	2.57	6.90	22.91	2.03	5.99	8.02
CDE-61	2.34	7.52	151.36	1.21	6.01	7.22
CDE-62	2.02	7.06	109.65	1.35	5.88	7.23
CDE-63	2.31	7.14	67.61	1.56	6.21	7.77
CDE-64	2.22	6.64	26.30	1.97	5.64	7.61
CDE-65	1.99	7.02	107.15	1.36	5.82	7.18
CDE-66	2.39	7.00	40.74	1.78	6.13	7.91
CDE-67	2.22	7.27	112.20	1.34	6.14	7.48
CDE-68	2.14	7.14	100.00	1.39	6.13	7.52
CDE-70	2.32	7.07	56.23	1.64	6.11	7.75
CDE-71	2.22	6.56	21.88	2.05	5.70	7.75
CDE-74	2.32	7.04	52.48	1.67	5.99	7.66
CDE-75	2.05	6.83	60.26	1.61	5.92	7.53
CDE-77	2.59	6.98	24.55	2.00	6.36	8.36
CDE-79	2.34	7.32	95.50	1.41	6.22	7.63
CDE-81	2.52	7.35	67.61	1.56	6.30	7.86
**Penta-CDE**						
CDE-82	3.30	7.38	12.02	2.31	6.30	8.61
CDE-85	3.16	7.44	19.05	2.11	6.28	8.39
CDE-87	3.04	7.70	45.71	1.73	6.51	8.24
CDE-89	3.04	7.23	15.49	2.20	6.11	8.31
CDE-90	2.83	7.64	64.57	1.58	6.54	8.12
CDE-91	2.83	7.24	25.70	1.98	6.06	8.04
CDE-97	3.00	7.48	30.20	1.91	6.22	8.13
CDE-99	2.87	7.61	54.95	1.65	6.38	8.03
CDE-100	2.66	7.33	46.77	1.72	6.11	7.83
CDE-101	2.76	7.56	63.10	1.59	6.22	7.81

CDE-102	2.69	7.12	26.92	1.96	5.98	7.94
CDE-105	3.29	7.67	23.99	2.01	6.51	8.52
CDE-108	3.00	7.83	67.61	1.56	6.52	8.08
CDE-109	3.00	7.86	72.44	1.53	6.58	8.11
CDE-110	2.91	7.35	27.54	1.95	6.31	8.26
CDE-114	3.15	8.06	81.26	1.48	6.61	8.09
CDE-115	2.83	7.77	87.10	1.45	6.47	7.92
CDE-116	2.76	7.94	151.36	1.21	6.37	7.58
CDE-117	2.80	7.76	91.20	1.43	6.41	7.84
CDE-118	3.02	7.83	64.56	1.58	6.60	8.18
CDE-119	2.74	7.62	75.83	1.51	6.44	7.95
CDE-120	2.74	8.06	208.93	1.07	6.66	7.73
CDE-123	3.02	7.89	74.13	1.52	6.63	8.15
CDE-126	3.25	8.25	100.00	1.39	6.83	8.22
**Hexa-CDE**						
CDE-128	4.06	8.14	12.02	2.31	6.82	9.13
CDE-130	3.73	8.55	66.07	1.57	7.01	8.58
CDE-132	3.75	7.96	16.22	2.18	6.47	8.65
CDE-137	3.70	8.44	54.95	1.65	6.72	8.37
CDE-138	3.77	8.31	34.67	1.85	7.01	8.86
CDE-139	3.46	8.47	102.33	1.38	6.84	8.22
CDE-140	3.57	8.10	33.88	1.86	6.65	8.51
CDE-146	3.42	8.41	97.92	1.40	6.67	8.16
CDE-147	3.42	8.42	100.00	1.39	6.76	8.15
CDE-149	3.37	7.88	32.36	1.88	6.47	8.35
CDE-153	3.46	8.36	79.43	1.49	6.72	8.21
CDE-154	3.19	8.04	70.79	1.54	6.49	8.03
CDE-156	3.87	8.78	81.28	1.48	7.07	8.55
CDE-157	3.93	8.49	36.31	1.83	6.99	8.82
CDE-163	3.50	8.30	63.10	1.59	6.78	8.37
CDE-166	3.64	8.94	199.53	1.09	6.95	8.04
CDE-167	3.64	8.72	120.23	1.31	7.11	8.42
**Hepta-CDE**						
CDE-170	4.42	9.12	50.12	1.69	7.28	8.97
CDE-174	4.14	8.89	56.12	1.64	6.98	8.62

CDE-177	4.23	9.09	72.44	1.53	7.14	8.67
CDE-180	4.20	9.50	199.53	1.09	7.46	8.55
CDE-181	4.17	9.64	295.12	0.92	7.31	8.23
CDE-187	3.92	9.05	134.89	1.26	7.13	8.39
CDE-189	4.33	9.54	162.18	1.18	7.55	8.73
CDE-190	4.22	9.46	173.78	1.15	7.31	8.46
**Octa-CDE**						
CDE-194	4.76	10.13	234.42	1.02	7.78	8.80
CDE-195	4.80	10.55	562.34	0.64	7.84	8.48
CDE-199	4.52	10.10	380.19	0.81	7.63	8.44
CDE-203	4.50	10.14	436.51	0.75	7.81	8.56
**Nona-CDE**						
CDE-206	5.16	11.45	1949.84	0.01	8.07	8.08
**Deca-CDE**						
CDE-209	5.80	12.95	14125.37	-0.76	8.16	7.40

Table S3. Summary of physicochemical properties of PCDEs predicted by QSPR models.

Compounds	logP$_L$ (Pa, 25 °C)					logK$_{ow}$ (25 °C)		logS$_{W,L}$ (mol/L, 25 °C)		
	Yang et al. [69]	Yuan et al. [73]	Zeng et al. A [72]	Zeng et al. B [72]	Sun et al. [71]	Yang et al. [69]	Sun et al. [71]	Yang et al. [69]	Xiao et al. [74]	Sun et al. [71]
DE	0.32	0.38	0.37	0.44	0.44	4.02	4.06	-3.28		-3.41
Mono-CDE	-0.38 to -0.33	-0.37 to -0.28	-0.49 to -0.28	-0.36 to -0.27	-0.30 to -0.27	4.48-4.61	4.38-4.65	-4.19 to -4.10		-4.29 to -4.01
Di-CDE	-1.08 to -0.88	-1.14 to -0.75	-1.16 to -0.55	-1.17 to -0.78	-1.10 to -0.87	4.81-5.21	4.69-5.25	-5.11 to -4.88		-5.35 to -4.61
Tri-CDE	-1.75 to -1.53	-1.88 to -1.31	-1.95 to -1.29	-1.79 to -1.35	-1.81 to -1.34	5.25-5.75	5.02-5.81	-5.97 to -5.71	-5.7	-6.30 to -5.38
Tetra-CDE	-2.42 to -2.03	-2.63 to -1.99	-2.52 to -1.86	-2.59 to -2.01	-2.58 to -1.97	5.46-6.28	5.33-6.36	-6.85 to -6.46	-6.4	-7.16 to -6.14
Penta-CDE	-3.04 to -2.71	-3.28 to -2.67	-3.15 ti -2.47	-3.19 to -2.65	-3.25 to -2.47	5.94-6.68	5.84-6.86	-7.74 to -7.38	-7.1	-8.18 to -7.06
Hexa-CDE	-3.67 to -3.35	-3.91 to -3.33	-3.75 to -3.24	-3.78 to -3.13	-3.90 to -2.90	6.43-7.08	6.33-7.35	-8.62 to -8.30	-7.9	-8.92 to -7.95
Hepta-CDE	-4.27 to -3.98	-4.40 to -4.04	-4.26 to -3.88	-4.33 to -3.85	-4.44 to -3.66	6.83-7.43	6.83-7.60	-9.49 to -9.24	-8.6	-9.68 to -9.00
Octa-CDE	-4.88 to -4.64	-4.91 to -4.61	-4.86 to -4.55	-4.88 to -4.44	-4.93 to -4.29	7.31-7.79	7.32-7.86	-10.38 to -10.13		-10.62 to -10.03
Nona-CDE	-5.43 to -5.31	-5.37	-5.47 to -5.32	-5.31 to -5.15	-5.30 to -5.02	7.80-8.07	7.82-8.08	-11.19 to -11.09		-11.38 to -11.19
Deca-CDE	-5.95	-5.83	-5.97	-5.75	-5.63	8.21	8.31	-12.03		-12.32

Table S4. Summary of toxic effects of PCDEs in organisms.

Compounds	Target	Methods	Adverse effects	Ref.
CDE-3, 7, 28 and 74	Trout	*In vivo*	Mortality	[45]
CDE-15	Green algae	*In vivo*	Photosynthesis inhibition of algal cells; Oxidative stress	[104]
CDE-77	Rainbow trout	*In vivo*	Increased EROD activity in liver	[109]
CDE-71, 77, 105 and 118	Japanese medaka	*In vivo*	Embryotoxicity; Mortality; Vascular hemorrhage	[50]
CDE-37, OH-CDE-37 and MeO-CDE-37	Crucian carp	*In vivo*	Oxidative stress in the liver at environmentally relevant concentrations	[75]
CDE-70	Zebrafish	*In vivo*	Altered vitellogenin content and related gene (vtg1) expression; Organelle damage in the liver and ovary;	[107]
CDE-15; OH-CDE-15	Zebrafish	*In vivo*	Mortality; Teratogenicity; Growth inhibition	[49]
CDE-77, 105, 126, 128, 157, 170, 177, 180, 187, 195, 203 and 207	Rat	*In vivo*	Increased activity of hepatic cytochrome P-450 and monooxygenase	[110]
CDE-1, 2 and 3	Rat	*In vivo*	Elevated hepatic mixed function oxidase (MFO) activities; Minor changed morphology of liver and thyroid	[105]
CDE-99, 153 and 184	Rat	*In vivo*	Elevated hepatic MFO activities; Minor changed morphology of liver, thymus and thyroid	[106]
CDE-77, 102 and 153	Rat	*In vivo*	Altered thyroid hormones	[53]
29 congeners [a]	H4IIE rat hepatoma cells	*In vitro*	Increased cytochrome P-450 1A1-associated enzyme activity and EROD activity	[108]
CDE-71, 77, 93, 100, 102, 118, 126, 153 and 154	Mice	*In vivo*	Reduced numbers of litters born and the survival of pups; Induced of cytochromes P-450	[52]
CDE-15 and OH-CDE-15	Mice	*In vivo*	Hepatic oxidative stress; Affected the balance of trace elements in liver	[51]
CDE-206, 207, 208 and 209	Mice	*In vivo*	Immunosuppressive activity; Induction of monooxygenase activity, ethoxyresorufin-O-deethylase (EROD) activity and levels of mRNA in liver	[54]

[a] CDE-47, 66, 77, 85, 99, 105, 118, 126, 128, 137, 138, 140, 147, 153, 154, 156, 157, 167, 170, 180, 181, 182, 190, 194, 195, 196, 197, 203 and 206.

Table S5. LC_{50} or EC_{50} values of PCDE congeners based on acute toxicity tests.

PCDE congeners	Target	Mean LC_{50} or EC_{50}(mg/L)					Ref.
		24h	48h	72h	96h	other	
CDE-3	Trout	1.40	1.00	0.84	0.73		[45]
CDE-7							
CDE-28							
CDE-74							
CDE-71	Japanese medaka					>2.5	[50]
CDE-77						0.17	
CDE-105						0.0108	
CDE-118						0.605	
CDE-37	Crucian carp				0.06		[75]
CDE-15	Zebrafish				1.45		[49]
DE	Zebrafish				5.01		[59]
CDE-3					1.19		
CDE-7					1.45		
CDE-15					1.00		
CDE-28					0.354		
CDE-30					0.562		
CDE-37					0.121		
CDE-66					0.0812		
CDE-77					0.0498		
CDE-99					0.144		
CDE-118					0.101		
CDE-209					5.46		
DE	Water flea		2.73				[59]
CDE-3			0.468				
CDE-7			0.771				
CDE-15			0.435				
CDE-28			0.215				
CDE-30			0.245				
CDE-37			0.0332				
CDE-66			0.0690				
CDE-77			0.0183				
CDE-99			0.0883				
CDE-118			0.0604				
CDE-209			5.71				
DE	Green algae				2.77[a]		[59]
CDE-3					0.595[a]		
CDE-7					1.40[a]		
CDE-15					0.838[a]		
CDE-28					0.425[a]		
CDE-30					0.449[a]		
CDE-37					0.356[a]		
CDE-66					0.253[a]		
CDE-77					0.139[a]		
CDE-99					0.370[a]		
CDE-118					0.292[a]		
CDE-209					2.57[a]		

[a] The EC_{50} value of PCDE congeners from acute toxicity tests.
